# Anion-Binding Properties of Aliphatic Symmetric Squaramide Receptors

**DOI:** 10.1021/acsomega.3c09094

**Published:** 2024-02-09

**Authors:** Serap Mert, Özden Erdebil

**Affiliations:** † Department of Chemistry and Chemical Processing Technology, Kocaeli University, Kocaeli 41140, Turkey; ‡ Department of Polymer Science and Technology, Kocaeli University, Kocaeli 41001, Turkey; § Center for Stem Cell and Gene Therapies Research and Practice, Kocaeli University, Kocaeli 41001, Turkey

## Abstract

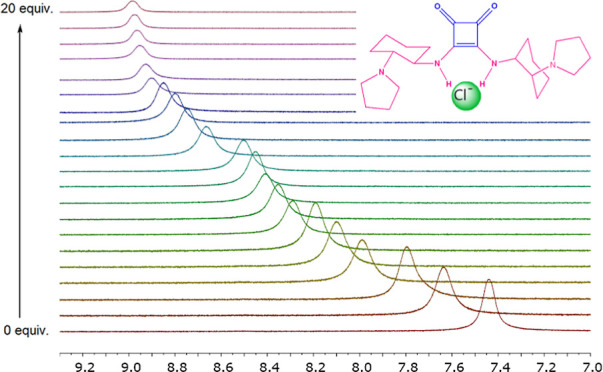

Squaramides (SQs), which are very popular for their H-bonding ability, have attracted great interest due to their wide range of applications such as asymmetric synthesis, pharmacology, and anion transportation. In this study, aliphatic symmetric SQs based on *cis*/*trans*-1,2-diaminocyclohexane (DACH) substituted with cyclic tertiary amines, synthesized in four steps under simple reaction conditions, were investigated for the first time for their ability to bind Cl^–^, Br^–^, and I^–^ anions. The changes in *cis*/*trans* geometric isomers and the cyclic ring (pyrrolidine vs piperidine) were found to have a combined effect on the degree of anion binding. The spectroscopic titrations of the SQs with TBA-Cl, TBA-Br, and TBA-I in the range of 0.2 to 20.0 equiv were monitored by ^1^H NMR, and the analyses of the magnitude of chemical shift differences in the NH peaks of the SQs in course of titration were performed by DynaFit and BindFit programs for the calculation of their *K*_a_ values. All symmetric SQs **I**–**IV** were found to selectively bind Cl^–^ anion more strongly than Br^–^ anion to varying degrees depending on the SQ derivatives. Especially, SQ **IV**, which has a symmetric *trans*-DACH and a pyrrolidine ring, was found to have the highest Cl^–^ anion-binding ability compared to the other SQs. However, the SQs did not show any change in the chemical shift of the NH proton in ^1^H NMR upon successive addition of TBA-I, indicating that they do not interact with I^–^ anion. The stoichiometries of the complexation behavior of SQs **I**–**IV** toward Cl^–^ and Br^–^ anions were also analyzed by Job plots.

## Introduction

The investigation of new receptors to monitor anions has become an active area of research because of environmental, chemical, physiological, and biological importance of anions.^1,2^ In particular, the roles of Cl^–^ anion in various cellular physiological processes such as membrane potential adjustment, pH setting, homeostasis, cellular differentiation, and cellular proliferation reveal the necessity of Cl^–^ anion for viability.^3−5^ Also, dysregulated Cl^–^ anions have been associated with some diseases like cystic fibrosis.^6^ Bromide is found in the saliva, serum, and urine of living organisms.^7,8^ Bromide deficiency results in hyperthyroidism, which impairs growth and fertility, while a high bromide level leads to bromide toxicity, or “bromism”, causing skin rashes. Iodide has been recognized as an essential micronutrient because of its important role in the development and functioning of the body.^8,9^ Found as an essential element in seawater, iodide is required for the synthesis of thyroid hormones in humans, and its deficiency causes goiter. Interactions between the anion and neutral or positively charged receptor like amide,^10,11^ sulfonamide,^12^ thio(urea),^13−18^ squaramide (SQ),^15,18−33^ and pyrrole, indole, and triazoles^34,35^ are usually hydrogen bonding^11,17,34−39^ and/or electrostatic, but in some cases, they result in deprotonation of the acidic hydrogen-bond donor.^17^ In particular, SQ derivatives^15,18−33^ exhibited high anion-attracting properties as they have favorable binding sites for anions due to two NH groups in their structure. Furthermore, aromatic groups on the NH of SQs increase the anion-binding ability of the molecule probably because of their ability of acting as additional H-bond donors of C(aryl)–H bonds.^19−26,28−32^

It has been shown in several studies that two NHs of SQs are good H-bond donors, allowing interaction of SQ-containing compounds with halides via hydrogen bonding.^15,18−33^ When Cl^–^ anion retention properties of SQs having the carbonyl functional group between two aromatic rings to act as molecular valves were investigated in nonpolar and polar solvents, ^1^H NMR and UV studies and density functional theory calculations showed that, in nonpolar solvents, carbonyls prevented Cl^–^ anion binding to SQ NHs through the intramolecular H-bonding of carbonyls with SQ NHs, but in polar solvents, disruption of the intramolecular H-bonding between carbonyl groups and SQ NHs provided space for the anion to bind SQs.^20^ When the interactions of various urea and SQ analogues with halogen anions (F^–^, Cl^–^, Br^–^, and I^–^) and oxoanions were compared, it was observed that the capacity of SQs to bind halogens was higher and the anion-binding constants decreased from F^–^ to I^–^ anions according to UV and ^1^H NMR titrations and X-ray crystal structure analysis.^15^ Similarly, Cl^–^ anion transport abilities of SQs were better than thio(ureas) through a series of unilamellar 1-palmitoyl-2-oleoylsn-glycero-3-phosphocholine (POPC) liposomes.^22^ Besides, Cl^–^ anion among various anions as a selective receptor enabled deprotonation of the NH proton of SQs containing anthracene and phenyl with different electron-withdrawing substituents (e.g., one or two trifluoromethyl and nitro groups), directly related to the degree of acidity due to the H-bond donor ability.^23^ More recent studies have shown that the interaction between Cl^–^, Br^–^, and nitrate (NO_3_^–^) anions and anthracene-conjugated SQs with –CF_3_ and –NO_2_ substituents, when analyzed computationally by energy decomposition analysis, is governed by a predominantly noncovalent process that generates attractive interaction energy consisting mainly of electrostatic energy and partly of orbital contribution.^29,32^

**Figure 1:**
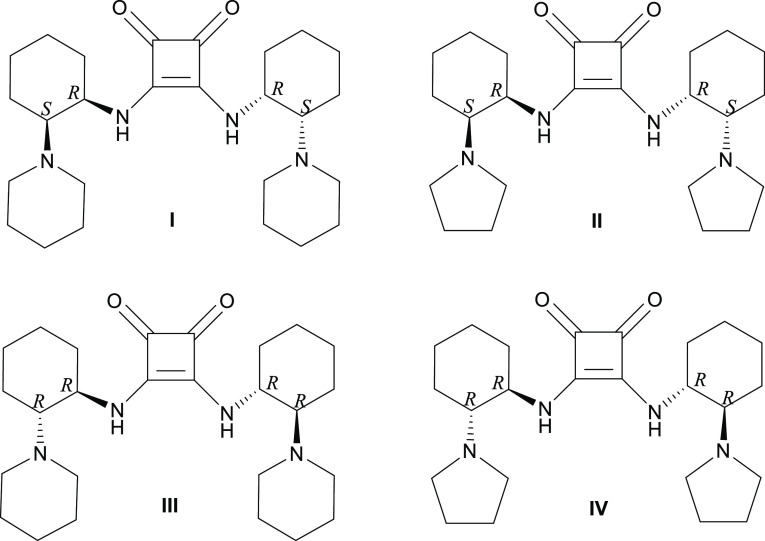
Structures of SQs **I**–**IV**.

Despite extensive anion-binding reports on SQs^15,18−33^ with aromatic substituents, to the best of our knowledge, no systematic report on the investigation of the binding properties of anions on SQs with only aliphatic substitutions has been attempted before. The presence of aromatic substituents in the NH group in SQs may increase the anion-binding capacity;^15,18−33^ however, anions might cause deprotonation by detaching protons from the NH of SQs depending on the substituent of the aromatic ring,^20,24,28^ and their low solubility both in some organic solvents^20,24^ and in water^28^ leads to a lack of understanding of their actual effects against cells in *in vitro* studies.^28^ In the current study, after the syntheses of symmetrical aliphatic SQs with *cis*-DACH **I** and **II** and *trans*-DACH **III** and **IV** moieties (Figure 1), three of which (**I**, **II**, and **IV**) were newly prepared by us, were carried out straightforward from *cis*- or *trans*-DACH in four steps, their Cl^–^, Br^–^, and I^–^ anion-binding properties were investigated for the first time in the literature. The presence of a pyrrolidine or piperidine ring together with *cis*- or *trans*-DACH geometric isomers in the structure is expected to cause the different molecular cavity and acidity (p*K*_a_)^40^ consequently affecting the anion-binding capabilities of aliphatic SQs to different degrees.^41^ In order to monitor the anion-binding abilities of SQs **I**–**IV**, ^1^H NMR spectroscopic titration was performed with TBA-Cl, TBA-Br, and TBA-I salts (guests) separately as the source of Cl^–^, Br^–^, and I^–^ anions. The results of spectroscopic titrations of SQs **I**–**IV** with TBA-Cl and TBA-Br were evaluated by the DynaFit and BindFit programs for the calculation of their association or binding constants (*K*_a_) because titration of the SQs with TBA-I did not show any chemical shift in the NH peaks. Furthermore, Job plots of the complexation behavior of SQs **I**–**IV** toward Cl^–^ and Br^–^ anions revealed that, depending on their geometry and cyclic substituents, SQs **I**–**IV** receptors exhibited variable binding abilities toward Cl^–^ and Br^–^ anions, but no interaction with I^–^ anion.

## Materials and Methods

### Materials

*cis*-1,2-Cyclohexane diamine (*cis*-DACH) (TCI, 97%), diallyl carbonate (Sigma-Aldrich, 99%), *Candida Antarctica* Lipase B (CAL-B, Novozym 435) (Strem Chemicals, 10000 PLU/g), potassium carbonate (K_2_CO_3_) (99%), 1,5-dibromopentane (Sigma-Aldrich, 97%), 1,4-diiodobutane (Sigma-Aldrich, 99%), palladium(II) acetate (Pd(OAc)_2_) (Sigma-Aldrich, 98%), 1,3-dimethyl barbituric acid (Sigma-Aldrich, 99%), triphenyl phosphine (PPh_3_) (Sigma-Aldrich, 99%), 3,4-dimethoxy-3-cyclo butene-1,2-dione (TCI, 98%), calcium chloride dihydrate (CaCl_2_·2H_2_O, Tekkim, 99%), sodium hydroxide (NaOH, Sigma-Aldrich), sodium chloride (NaCl, Sigma-Aldrich), HCl in methanol solution (Sigma-Aldrich, 3.2 M), di-*tert*-butyl dicarbonate (Boc_2_O) (TCI, 95%), and trifluoroacetic acid (Sigma-Aldrich, 99%) were used for the syntheses of *cis*/*trans*-DACH-based receptors. Absolute toluene (Sigma-Aldrich, 99.7%) was obtained by distillation over sodium using benzophenone as an indicator. Methanol (Sigma-Aldrich, 99.9%) was dried by distilling over magnesium. Absolute dichloromethane (Sigma-Aldrich, 99.9%), acetonitrile (Sigma-Aldrich, 99.9%), and 1,2-dichloroethane (Sigma-Aldrich, 99%) were prepared by distillation over calcium hydride. Tetrabutylammonium chloride (TBA-Cl, Sigma-Aldrich), tetrabutylammonium bromide (TBA-Br, TCI Europe), tetrabutylammonium iodide (TBA-l, TCI Europe), and [*d*_6_]DMSO (Eurisotop) were used for ^1^H NMR spectroscopic titration.

### Instruments

An ATR Bruker Tensor 27 spectrometer was used for ATR–FTIR analyses with a range of 4000–600 cm^–1^, a resolution of 4 cm^–1^, and 30 scans for each measurement. A Bruker AVANCE-III 400 MHz NMR spectrometer was used to characterize the synthesized intermediates and products and to perform spectroscopic titration. Mass analyses of novel *cis*/*trans*-DACH-based SQs were measured with 6200 series TOF/6500 series Q-TOF B.08.00 (B8058.0). Moisture-sensitive starting materials were weighed in a glovebag, and the reactions were continued under an argon atmosphere. Stuart brand SMP30 was used for measuring the melting points (MPs) of the solid intermediates and SQs.

## Methods

Syntheses and characterizations of compounds based on *cis*-DACH **1**–**5** and *trans*-DACH **6**–**10** were reported in our previous work.^42,43^

### Synthesis of 3,4-Bis(((1*R*,2*S*)-2-(piperidin-1-yl)cyclohexyl)amino)cyclobut-3-ene-1,2-dione (**I**)

Literature-known (1*R*,2*S*)-2-(piperidin-1-yl)cyclohexanamine (**4**)^42,43^ (276 mg, 1.514 mmol) and commercially available 3,4-dimethoxy-3-cyclobutene-1,2-dione (108 mg, 0.757 mmol) were stirred in dry dichloromethane (3 mL) under an argon atmosphere at RT for 51 h. The resulting crude substance was purified by using column chromatography with 140/10/1 (DCM/MeOH/Et_3_N), and then, it was crystallized with DCM/ether. Yield: 174 mg (52%). MP >160.4 °C with decomposition. ATR–FTIR (*v*_max_/cm^–1^): 3150 (br, NH), 1791 (s, C=O), 1664, 1188, 1111, 870, 698. ^1^H NMR (400 MHz, [*d*_6_]DMSO): δ 7.47 (s, 2H), 4.59 (s, 2H), 2.54 (s, 2H), 2.46 (s, 4H), 2.27 (s, 2H), 1.82 (m, 6H), 1.59–1.20 (m, 24H). ^13^C NMR (101 MHz, [*d*_6_]DMSO): δ 183.21, 168.91, 64.60, 50.94, 50.79, 50.66, 32.35, 26.44, 25.31, 24.86, 23.93, 19.69. HRMS: C_26_H_42_N_4_O_2_ ([M + H]^+^) calcd, 443.3381; found, 443.33784.

### Synthesis of 3,4-Bis(((1*R*,2*S*)-2-(pyrrolidin-1-yl)cyclohexyl)amino)cyclobut-3-ene-1,2-dione (**II**)

(1*R*,2*S*)-2-(Pyrrolidin-1-yl)cyclohexanamine (**5**)^42,43^ (100 mg, 0.594 mmol) and 3,4-dimethoxy-3-cyclobutene-1,2-dione (42 mg, 0.297 mmol) were mixed in dry dichloromethane (2 mL) under an argon atmosphere at RT for 51 h. The resulting crude substance was purified by using column chromatography with 140/10/1 (DCM/MeOH/Et_3_N), and then, the crystallization was performed with DCM. Yield: 65 mg (53%). MP >191 °C with decomposition. ATR–FTIR (*v*_max_/cm^–1^): 3150 (br, NH), 1791 (s, C=O), 1664, 1188, 1111, 870, 698. ^1^H NMR (400 MHz, [*d*_6_]DMSO): δ 7.55 (d, *J* = 8.2 Hz, 2H), 4.44 (s, 2H), 2.11 (d, *J* = 11.1 Hz, 3H), 1.83 (d, *J* = 13.0 Hz, 5H), 1.67 (m, 12H), 1.30–151 (m, 14H). ^13^C NMR (101 MHz, [*d*_6_]DMSO): δ 182.28, 167.83, 64.91, 51.09, 31.61, 25.80, 24.04, 22.84, 19.29. HRMS: C_26_H_42_N_4_O_2_ ([M + H]^+^) calcd, 415.3068; found, 415.3062.

### Synthesis of 3,4-Bis(((1*R*,2*R*)-2-(piperidin-1-yl)cyclohexyl)amino)cyclobut-3-ene-1,2-dione (**III**)^44^

(1*R*,2*R*)-2-(Piperidin-1-yl)cyclohexanamine (**9**)^42,43^ (175 mg, 0.96 mmol) and 3,4-dimethoxy-3-cyclobutene-1,2-dione (68 mg, 0.48 mmol) were mixed in dry dichloromethane (3 mL) and stirred under an argon atmosphere at RT for 54 h. After the reaction solvent was removed, the resulting crude substance was purified by crystallization with diethyl ether. Yield: 136 mg (64%). mp >272 °C with decomposition. ATR–FTIR (*v*_max_/cm^–1^): 3186 (br, NH), 1795 (s, C=O), 1635, 1188, 1105, 863, 742. ^1^H NMR (400 MHz, [*d*_6_]DMSO): δ 7.18 (d, *J* = 8.7 Hz, 2H), 3.79 (d, *J* = 10.6 Hz, 2H), 2.60 (s, 4H), 2.28 (s, 6H), 2.00 (d, *J* = 11.6 Hz, 2H), 1.84–1.60 (m, 6H), 1.44–1.13 (m, 20H). ^13^C NMR (101 MHz, [*d*_6_]DMSO): δ 182.78, 168.77, 68.01, 52.24, 51.13, 48.88, 34.41, 24.33, 23.82, 23.10, 22.74, 21.62.

### Synthesis of 3,4-Bis(((1*R*,2*R*)-2-(pyrrolidin-1-yl)cyclohexyl)amino)cyclobut-3-ene-1,2-dione (**IV**)

(1*R*,2*R*)-2-(Pyrrolidin-1-yl)cyclohexanamine (**10**)^43^ (150 mg, 0.891 mmol) and 3,4-dimethoxy-3-cyclobutene-1,2-dione (63 mg, 0.445 mmol) were stirred in dry dichloromethane (3 mL) under an argon atmosphere at RT for 54 h. After the reaction solvent was removed, the resulting crude substance was purified by crystallization with ethanol. Yield: 114 mg (62%). MP >206 °C with decomposition. ATR–FTIR (*v*_max_/cm^–1^): 3149 (br, NH), 1797 (s, C=O), 1633, 1139, 1010, 868, 742. ^1^H NMR (400 MHz, [*d*_6_]DMSO): δ 7.43 (d, *J* = 8.4 Hz, 2H), 3.92 (s, 2H), 2.60 (d, *J* = 7.8 Hz, 4H), 2.45 (s, 2H), 1.98 (s, 2H), 1.81–1.51 (m, 16H), 1.41–1.17 (m, 10H). ^13^C NMR (101 MHz, [*d*_6_]DMSO): δ 181.92, 167.58, 62.60, 54.28, 47.93, 32.37, 23.39, 23.15. HRMS: C_26_H_42_N_4_O_2_ ([M + H]^+^) calcd, 415.3068; found, 415.30625.

### NMR Titration Experiments

For the titrations, a 0.055 M solution of the receptors in [*d*_6_]DMSO was prepared first as a host solution. Then, a 1 M solution of the TBA-Cl salt in [*d*_6_]DMSO as an anionic guest was added in aliquots starting 0.2 up to 20 equiv with respect to the receptors. After each anion addition, ^1^H NMR spectra were taken, and the changes in the H peak of NH were monitored for each receptor. The titration protocol of SQs **I**–**IV** with TBA-Cl was also applied for TBA-Br and TBA-I, respectively.

## Results and Discussion

### Synthesis of SQs

**Scheme 1:**
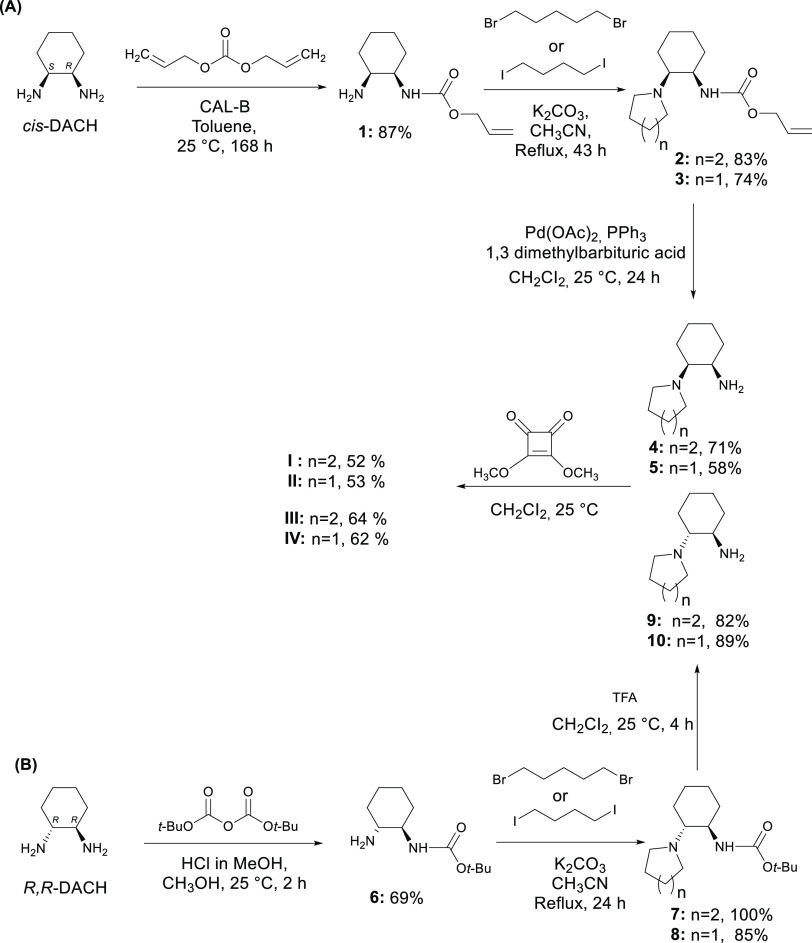
Synthesis of (A) *cis*- and (B) *trans*-DACH-Based SQ Receptors **I**–**IV**

SQs **I** and **II** with the *cis*-DACH skeleton were obtained in a four-step reaction,^42,45^ as described sequentially below, with yields in each step between 52 and 87% and with total yields in 27 and 20%, respectively (Scheme 1A): (i) the formation of mono-*N*-allyloxycarbonyl (Alloc) protected compound **1** by desymmetrization of the meso compound, *cis*-DACH, by CAL-B with diallyl carbonate in toluene, (ii) the reaction of compound **1** with 1,5-dibromopentane or 1,4-diiodobutane in the presence of K_2_CO_3_ to give the compounds **2** and **3** with piperidine or pyrrolidine as a cyclic tertiary amine, respectively, (iii) deprotection of the Alloc group from compounds **2** and **3** by exploiting palladium diacetate and 1,3-dimethylbarbituric acid to yield compounds **4** and **5**, and (iv) formation of *cis*-isomer SQs **I** and **II** receptors by the coupling of **4** and **5** with 3,4-dimethoxy-3-cyclobutene-1,2-dione (Scheme 1A).

Receptor SQs **III** and **IV** with the *trans*-DACH moiety were obtained in the following steps^42,46^ in total yields of 36 and 32%, respectively, when started from *R,R*-DACH (Scheme 1B): (i) the reaction of *R,R*-DACH with di-*tert*-butyl dicarbonate (Boc_2_O) in acidic medium to afford mono-Boc-protected DACH **6**, (ii) the cyclization of free amine of the compound **6** with 1,5-dibromopentane or 1,4-diiodobutane in the presence of a base, K_2_CO_3_, to give a piperidine or pyrrolidine ring, respectively, for the synthesis of compounds **7** and **8**, (iii) the deprotection of the Boc group in the intermediates **7** and **8** in the presence of trifluoroacetic acid to give the compounds **9** and **10**, and (iv) the formation of SQs **III**(44) and **IV** receptors by the coupling of **9** and **10** with 3,4-dimethoxy-3-cyclobutene-1,2-dione.

The syntheses and characterizations of compounds based on *cis*-DACH **1**–**5** and *trans*-DACH **6**–**10** starting from corresponding amines were previously reported by us^42,43^ and others.^45,46^*cis*- and *trans*-DACH-based receptor SQs **I**, **II**, **III**, and **IV** were characterized fully by ATR–FTIR and ^1^H and ^13^C NMR analyses (Supporting Information, Figures S1–S3, S5–S7, S9–S14). Moreover, the successful syntheses of novel DACH-based receptors with *cis*-isomer SQs **I** and **II** and *trans*-isomer SQ **IV** were confirmed by mass analyses (Supporting Information, Figures S4, S8, and S15).

### ^1^H NMR Titrations of SQs with TBA-Cl, TBA-Br, and TBA-I

**Figure 2:**
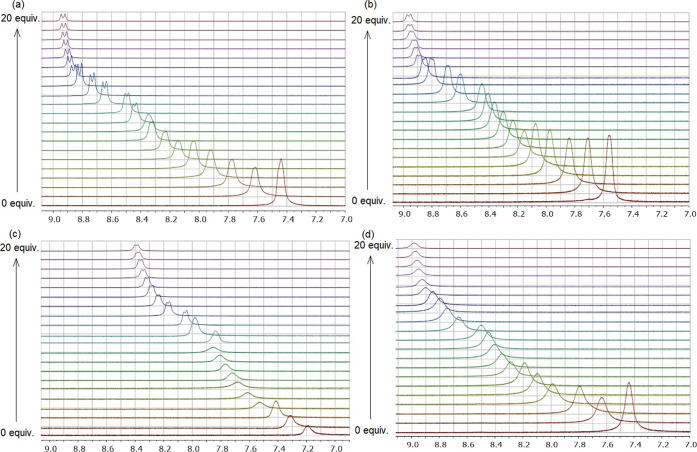
Stack plots of ^1^H NMR spectra of addition of TBA-Cl (0–20 equiv) in <0.02% H_2_O in [d_6_] DMSO at 25 °C to SQ **I** (a), SQ **II** (b), SQ **III** (c), and SQ **IV** (d).

The spectroscopic titrations of SQs **I**–**IV** with TBA-Cl, TBA-Br, or TBA-I in the equivalent range of 0.2 to 20.0 were monitored by ^1^H NMR, and the differences in the chemical shift in NH peaks of SQs upon successive addition of anions during titration were noted. The spectra for the titration of SQs **I**–**IV** with TBA-Cl are shown in Figure 2. The downfield shift of the SQ protons indicates H-bond formation between Cl^–^ anion and NHs of all SQs **I**–**IV**. The peak of NH protons of SQs **I** and **III** containing a piperidine ring shifted from 7.47 and 7.17 ppm to 8.94 and 8.38 ppm in ^1^H NMR, respectively, after the addition of 20 equiv of the TBA-Cl salt, as indicated in Figure 2a,c. The only difference between SQs **I** and **III** with a piperidine ring is that SQ **I** has a *cis*-DACH moiety, while SQ **III** has a *trans*-DACH moiety. The difference in the chemical shift was 1.47 ppm for SQ **I** with *cis* geometry and 1.21 ppm for SQ **III** with *trans* geometry in the ^1^H NMR titration. It can be concluded that the *cis*-DACH skeleton with *S,R* conformation and a piperidine ring in SQ **I** displays higher affinity for Cl^–^ anion when compared to the diastereomeric isomer of SQ **III**. Moreover, SQ **II** with the *cis*-DACH moiety and SQ **IV** with the *trans*-DACH moiety, both containing a pyrrolidine ring, were compared, and the protons of NH shifted downfield by 1.38 ppm (from 7.57 to 8.95 ppm) for SQ **II** and by 1.55 ppm (from 7.43 to 8.98 ppm) for SQ **IV** when both were titrated with 20 equiv of the TBA-Cl salt (Figure 2b,d). In other words, the difference in the chemical shift of protons of NHs of SQs in the ^1^H NMR as a result of titration was ordered as SQ **IV** > **I** > **II** > **III**. It can be concluded that the *trans*-DACH skeleton with the *R,R* conformation and pyrrolidine ring in SQ **IV** provides higher affinity for Cl^–^ anion when compared to the diastereomeric isomer of SQ **II**. Overall, all results indicate that SQ **IV** with the *trans*-DACH moiety and pyrrolidine ring and SQ **I** with the *cis*-DACH moiety and piperidine ring have much better Cl^–^ anion retention than SQ **II** with the *cis*-DACH moiety and pyrrolidine ring and SQ **III** with the *trans*-DACH moiety and piperidine ring, respectively. This also means that the H-bond strengths were high in *cis*-SQ **I** and *trans*-SQ **IV**. It was concluded that both *cis*/*trans*-DACH moiety and the piperidine/pyrrolidine ring were active in the ability of Cl^–^ anion binding. This can be attributed to the difference in the cavity size formed by the placement of axial and equatorial positions of the *cis*- and *trans*-DACH geometry and in the degree of acidity of the molecule resulting from the piperidine and pyrrolidine ring moiety.

**Figure 3:**
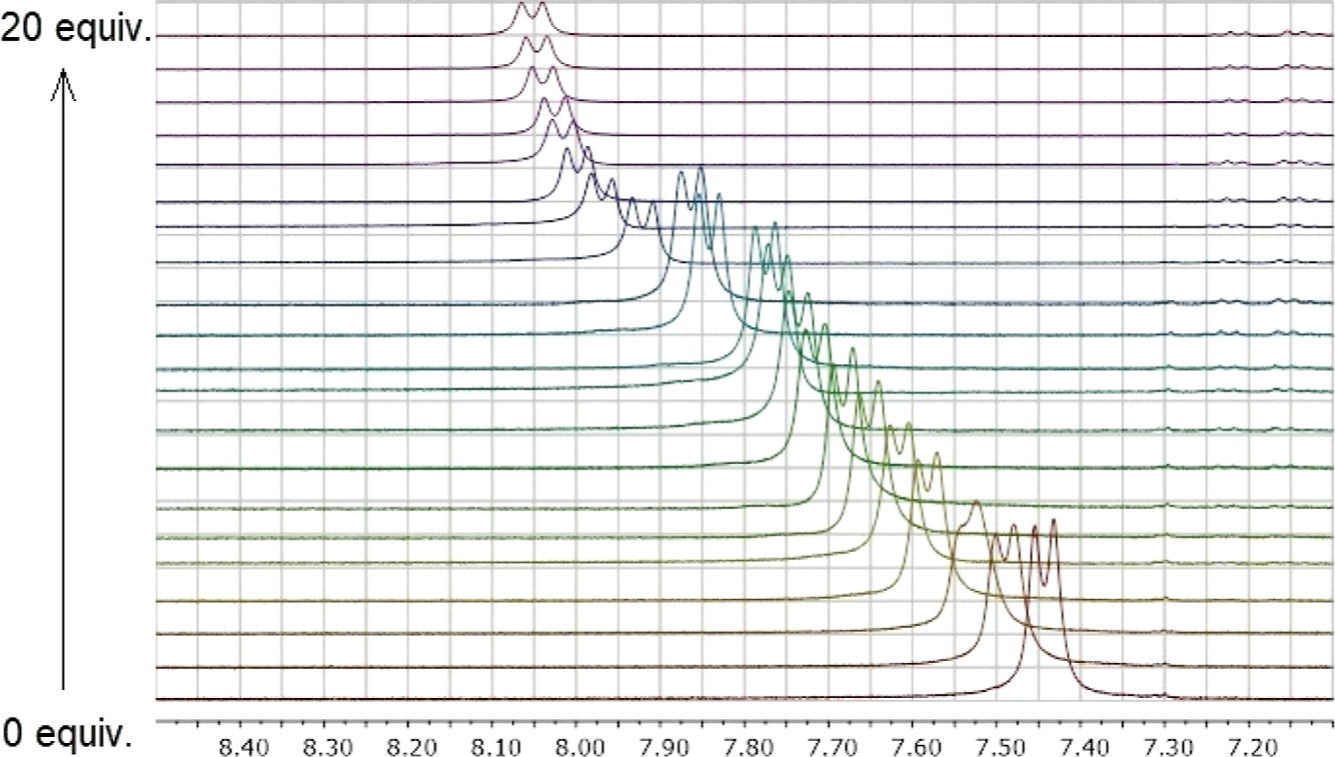
Stack plots of ^1^H NMR spectra of addition of TBA-Br (0–20 equiv) in <0.02% H_2_O in [d_6_] DMSO at 25 °C to SQ **I**.

For the titration of SQs **I**–**IV** with TBA-Br, the downfield shifts of NH protons were observed upon the successive addition of TBA-Br in ^1^H NMR, as shown in Figure 3 and Supporting Information, Figures S16–S18. The highest shift difference was obtained as 0.61 ppm (from 7.43 to 8.04 ppm) for SQ **I** with the *cis* geometry and piperidine ring and 0.56 ppm for SQ **IV** with the *trans* geometry and pyrrolidine ring (from 7.47 to 8.03 ppm) (Figure 3 and Supporting Information, Figure S18). SQ **II** with the *cis* geometry and pyrrolidine ring (0.49 ppm; 7.57 to 8.06 ppm) and SQ **III** with the *trans* geometry and piperidine ring (0.36 ppm, 7.17 to 7.53 ppm) are third and fourth in the order of chemical shift difference in ^1^H NMR titration (Supporting Information, Figures S16 and S17), respectively. It can be concluded that the differences in NH shift values in SQs are in the order of SQ **I** > **IV** > **II** > **III** as a result of their titration with TBA-Br in ^1^H NMR.

**Figure 4:**
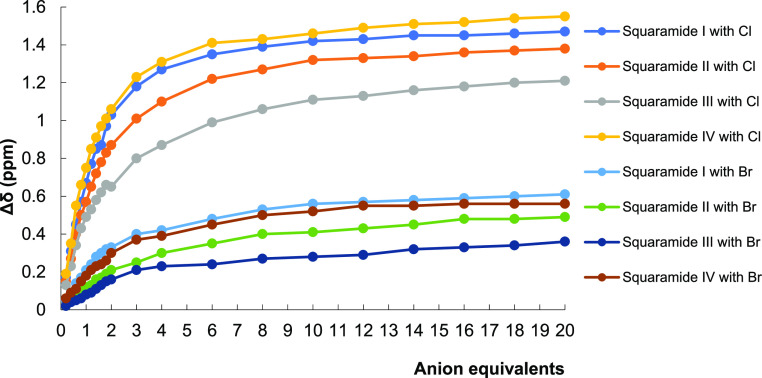
Chemical shift difference of the NH proton with the successive addition of TBA-Cl and TBA-Br equivalent.

A graphical representation of all changes in chemical shifts (Δδ) against the equivalents of Cl^–^ and Br^–^ anions in ^1^H NMR spectroscopic titrations is also demonstrated in Figure 4. Comparing the data for Br^–^ anion with those for Cl^–^ anion, it is seen that the magnitudes of SQs **I** and **IV** are larger than that of SQs **II** and **III** for both anions, although the order of SQs **I** and **IV** changes. It indicates that SQ **I** with the *cis*-DACH moiety and piperidine ring and SQ **IV** with the *trans*-DACH moiety and pyrrolidine ring have much better Br^–^ anion retention than SQ **II** with the *cis*-DACH moiety and pyrrolidine ring and SQ **III** with the *trans*-DACH moiety and piperidine ring, respectively. As a result, when the behavior of SQs **I**–**IV** toward Cl^–^ anion is compared to that of Br^–^ anion, the difference in chemical shifts becomes very apparent that SQs are more selective toward Cl^–^ anion. This can be explained by the fact that Cl^–^ anion, which has a larger electron density, is better recognized by the receptors than Br^–^ anion, as reported in the literature.^32^ Besides, different Δδ values ranging from 1.21 to 1.55 ppm for the NH signal against Cl^–^ and 0.36–0.61 ppm for the NH signal against Br^–^ point out the distinct nature and strength of H-bond between SQs **I**–**IV** and different anions.^26,30^ On the other hand, titration of the SQs with TBA-I did not show any difference in the chemical shift of NH peaks in ^1^H NMR spectroscopy. Therefore, it has even been found that SQs **I**–**IV** do not show any affinity toward I^–^ anion.

### Binding Properties of SQs

The chemical shifts in the NH peaks obtained from ^1^H NMR titration of SQs with TBA-Cl or TBA-Br were also analyzed by DynaFit^47−49^ and BindFit^50−52^ programs according to the 1:1 model. In addition, the stoichiometries of the complexation behaviors of SQs **I**–**IV** toward Cl^–^ or Br^–^ anions were analyzed by Job plots.^52−54^

Fitplots of downfield shifts of NH protons vs the concentration of TBA-Cl (Supporting Information, Figure S19a,d,g,j) or TBA-Br (Supporting Information, Figure S20a,d,g,j), residual for TBA-Cl (Supporting Information, Figure S19b,e,h,k) and for TBA-Br (Supporting Information, Figure S20b,e,h,k), and the relative sum of squares (SSQ/SSQ min.) for TBA-Cl (Supporting Information, Figure S19c,f,i,l) and for TBA-Br (Supporting Information, Figure S20c,f,i,l) were acquired from the DynaFit program by fitting a 1:1 model for SQs **I**–**IV** and Cl^–^ or Br^–^ anion binding. The random appearance of the residual graph from the DynaFit program proves that the data is useable and compatible with the program.^53^ The program evaluates all possible combinations and determines the number of independent least-squares correspondingly.^47^ The residuals (Supporting Information, Figures S19b,e,h,k and S20b,e,h,k), sum of squares (Supporting Information, Figures S19c,f,i,l and S20c,f,i,l), and fitplots mentioned above for SQs **I**–**IV** with Cl^–^ or Br^–^ anions were supportive data for the *K*_a_ value with a high confidence interval of 95% from the DynaFit software. One representative example of the DynaFit script is given in detail in the Supporting Information.

As a result of the analyses of the titration data with DynaFit, the stoichiometric *K*_a_ values for Cl^–^ anion as 25.61 M^–1^ for SQ **I**, 18.02 M^–1^ for SQ **II**, 12.31 M^–1^ for SQ **III**, and 27.34 M^–1^ for SQ **IV** and for Br^–^ anion as 11.08 M^–1^ for SQ **I**, 4.65 M^–1^ for SQ **II**, 5.89 M^–1^ for SQ **III**, and 8.25 M^–1^ for SQ **IV** were calculated according to the 1:1 model,^47,50,53^ as summarized in Table 1. Therefore, the order of binding tendencies of SQs in terms of the *K*_a_ value was found to be SQ **IV** > **I** > **II** > **III** for Cl^–^ and SQ **I** > **IV** > **III** > **II** for Br^–^. A higher H-bond strength in SQs **I** and **IV** is probably due to strong interaction of Cl^–^ or Br^–^ anion with hydrogens of SQs in the most favorable cavity formed by their structures. Moreover, the *K*_a_ values calculated using DynaFit and the H-bond strengths observed by the shift difference in ^1^H NMR titration confirm each other. Our previous findings revealed that the SQs as organocatalysts are conjugated to each other by intramolecular H bonds from NH and carbonyl groups of SQs.^42^ This high self-bonding tendency of SQs can also complicate the study of anion bonding especially in solvents behaving as a good H-bond acceptor, such as DMSO. Despite this self-binding tendency leading to a medium *K*_a_ value, it is very important to show that SQs **I** and **IV** can strongly bind with Cl^–^ anion according to the 1:1 binding model.

**Table 1:** Binding Constants (*K*_a_ Values) of SQs **I**–**IV** Calculated According to the DynaFit 1:1 and BindFit 1:1 Software

	*K*_a_ (M^–1^) ± S.D.				
anion	model	SQ **I**	SQ **II**	SQ **III**	SQ **IV**
Cl^–^	DynaFit 1:1	25.61 ± 0.89	18.02 ± 0.36	12.31 ± 1.00	27.34 ± 1.20
Cl^–^	BindFit 1:1	24.63 ± 3.20	18.02 ± 1.54	14.13 ± 6.05	27.34 ± 4.07
Br^–^	DynaFit 1:1	11.08 ± 0.75	4.65 ± 0.31	5.89 ± 0.98	8.25 ± 0.54
Br^–^	BindFit 1:1	11.14 ± 4.13	4.63 ± 2.56	5.49 ± 7.27	8.64 ± 3.56

SQs **I**–**IV** interact more strongly with Cl^–^ anion than with Br^–^ anion, while they do not interact with I^–^ anion. The BindFit program, which is frequently used in supramolecular anion-binding studies, was also used to analyze the data. Binding constants (*K*_a_ values with standard deviations) of SQs **I**–**IV** calculated according to the DynaFit^47−49^ and BindFit^50,52^ software with model 1:1 are summarized in Table 1. Overall, *K*_a_ values in Table 1 indicate that SQ **I** with *cis*-DACH/piperidine has much better Cl^–^ anion retention than SQ **III** with *trans*-DACH/piperidine, whereas SQ **IV** with *trans*-DACH/pyrrolidine displays better performance than SQ **II** with *cis*-DACH/pyrrolidine. It reveals that the *K*_a_ value of SQs for anions depends not only on the *cis*/*trans* configuration but also on the substituents of the piperidine/pyrrolidine ring. The highest *K*_a_ value (27.34 M^–1^) calculated by both the DynaFit and BindFit programs for SQ **IV** and Cl^–^ anion indicates that SQ **IV** forms the strongest H-bond formation with Cl^–^ anion among other SQs (**I**–**III**). The analyses of the BindFit program are provided in the Supporting Information (Figures S21–S28). The fact that the *K*_a_ values obtained from both programs are very close to each other can be considered as supportive results, in terms of the validity of both programs. Since the titrations of SQs **I**–**IV** with TBA-I did not show any chemical shift in the NH peaks in ^1^H NMR spectroscopy, *K*_a_ values were not calculated.

### Job Plots

**Figure 5:**
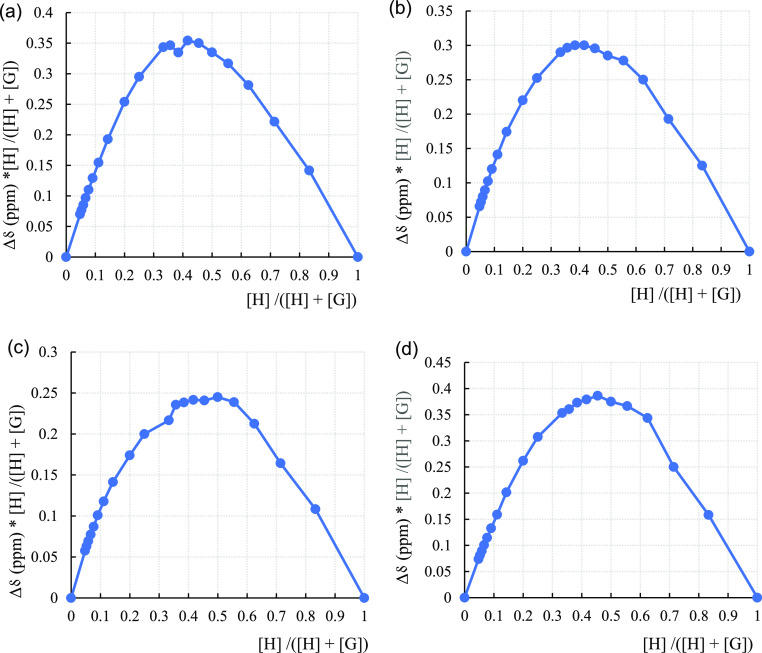
Job plots^54^ of ^1^H NMR spectroscopic TBA-Cl titration with SQ **I** (*X*_max_: 0.42) (a), SQ **II** (*X*_max_: 0.42) (b), SQ **III** (*X*_max_: 0.5) (c), and SQ **IV** (*X*_max_: 0.45) (d).

The maximum host fraction (*X*_max_) for SQs **I**, **II**, **III**, and **IV** in the presence of TBA-Cl was found as 0.42, 0.42, 0.5, and 0.45 from the Job plots, respectively (Figure 5). Then, the host/guest (H/G) stoichiometric ratio was 1:1 (XY) from the formula *Y* = (1/*X*_max_) – 1 for SQ **III**. Regarding the titration of SQs **I**, **II**, and **IV**, both *XY* and *XY*_2_ complex formation occurred in the medium when their *X*_max_ values were considered.^53^ Similarly, 1:1 and 1:2 composite models were obtained for both SQ **I** (*X*_max_: 0.42) and SQ **III** (*X*_max_: 0.38) along with 1:2 for both SQs **II** and **IV** (*X*_max_: 0.33) when the titrations of the SQs with TBA-Br were analyzed (Supporting Information, Figure S29). As a result, DynaFit 1:1 and BindFit 1:1 were used to verify the *K*_a_ values, and the Job plot was used to correlate the binding stoichiometry. In particular, the SQs show that the 1:1 or composite binding stoichiometry (1:1 + 1:2) model binds Cl^–^ anion, and the composite 1:1 + 1:2 or only 1:2 model binds Br^–^ anion.

The actual stoichiometries, which are more easily influenced by the host, were the same/very close to the Job plot or in some cases slightly lower^52^ than the Job plot in the present study. Although it is claimed in the literature that the Job plot is still valid for the analysis of inorganic complexes,^53^ some studies in the literature suggest that the data should be tested and systematically fitted to all possible binding models because the Job plot is not suitably good for host–guest supramolecular chemistry.^52,53^ In fact, if there is any uncertainty for the stoichiometry binding model or the correct stoichiometry, it is suggested that the raw data should be examined with all possible models, and the results should be compared.

## Conclusions

We synthesized nonaromatic symmetrical SQs with *cis* and *trans* moieties to investigate their ability to bind Cl^–^, Br^–^, and I^–^ anions. The downfield shift of protons of –NH in the course of titrations in ^1^H NMR indicates that H-bonding interactions occur between NH protons of SQs and more significantly Cl^–^ or less apparently Br^–^ anions. The reason for this result, as reported in the literature,^32^ is that Cl^–^ anion, which has a larger electron density, is better recognized by the receptors than Br^–^ anion. The data were analyzed by the Job plot to examine the stoichiometric value, and open access DynaFit and BindFit programs, which is a very popular method for supramolecular studies, were used for the calculation of *K*_a_ values of these SQs with Cl^–^ or Br^–^ anions. As a result of analyses of spectroscopic titration data by both DynaFit and BindFit software, SQ **IV** with a symmetrical *trans*-DACH structure and pyrrolidine ring was found to have the highest Cl^–^ anion-binding ability. The overall order of *K*_a_ values of SQs for Cl^–^ was found to be **IV** > **I** > **II** > **III**. It was also determined that Cl^–^ anion made much stronger H bonds with the SQs than Br^–^ anion according to the *K*_a_ values obtained, while there was no interaction between SQs and I^–^ anion. It also reveals that geometrical (i.e., *cis* or *trans*) or structural (i.e., pyrrolidine or piperidine ring) differences affect the anion-binding capability to different extents. This can be attributed to the differences in the molecular cavity and p*K*_a_^40^ arising from positions of *cis*/*trans*-geometric isomers and the change in the ring size from pyrrolidine to piperidine. Based on the findings in this study, the potential anion-binding bioapplications of SQs **I**–**IV** could be further expanded in future studies.

## Supporting Information Available

^1^H NMR,^13^C NMR, ATR–FTIR, and LC/MS–TOF spectra of novel SQs, stack plots of ^1^H NMR spectra of addition of TBA-Br to SQs **I**–**IV**, example for 1:1 binding model DynaFit script, graphs of DynaFit 1:1 analysis, graphs of BindFit 1:1 analysis, and Job plots (PDF)
