# Supplementary material for: Anion-Binding Properties of Aliphatic Symmetric Squaramide Receptors
Source: ACS Omega. 2024 Feb 9;9(7):8333–42. doi: 10.1021/acsomega.3c09094 (PMC10883022; doi:10.1021/acsomega.3c09094)
Supplement: Supplementary file 1 — ao3c09094_si_001.pdf [file ao3c09094_si_001.pdf]

Supporting Information

Anion Binding Properties of Aliphatic Symmetric Squaramide Receptors

Serap Mert,[1-3] Özden Erdebil[2]*

[1] Department of Chemistry and Chemical Processing Technol., 41140, Kocaeli University, Turkey
[2] Department of Polymer Sci. and Technol., Kocaeli University, 41001, Kocaeli, Turkey
[3] Center for Stem Cell and Gene Therapies Res. and Pract., 41001, Kocaeli University, Turkey

Contents

I. ^{1}H NMR,^{13}C NMR, ATR-FTIR, and LC/MS-TOF spectra of squaramides S2
II. Stack plots of ^{1}H NMR spectra of addition of TBA-Br to SQs II-IV S12
III. Example 1:1 Binding Model DYNAFIT Script[1] S14
IV. Graphs of DynaFit 1:1 Analysis[1] S15
V. Graphs of BindFit 1:1 analysis[2, 3] S17
VI. Job plots[4] S21
REFERENCES S22

I. ^{1}H NMR, ^{13}C NMR, ATR-FTIR, and LC/MS-TOF spectra of squaramides

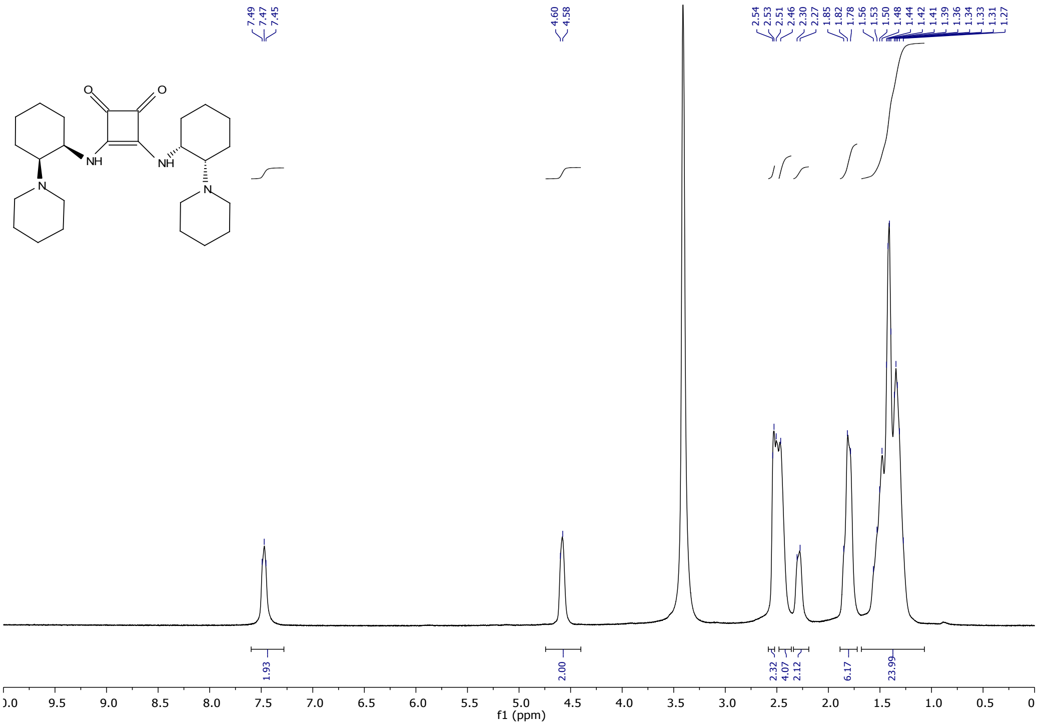

Figure S.1 ^{1}H-NMR Spectrum of 3,4-bis(((1R,2S)-2-(piperidin-1-yl)cyclohexyl) amino)cyclo but-3-ene-1,2-dione (**I**)

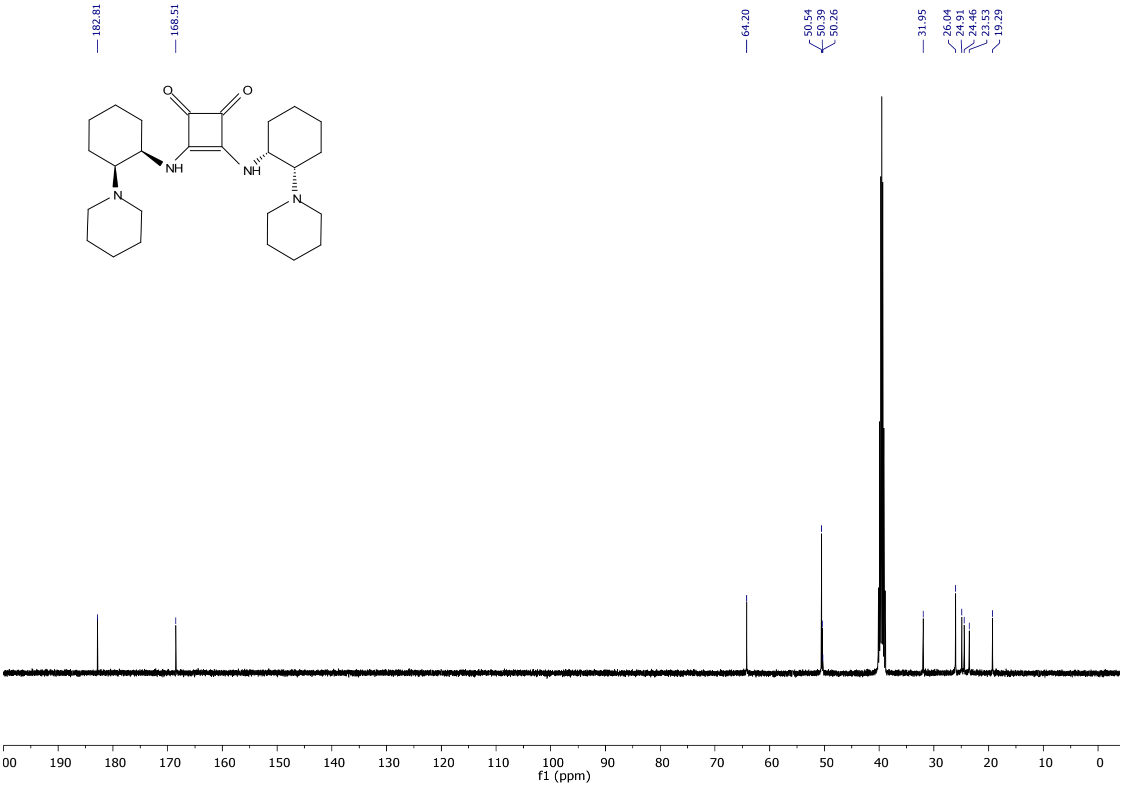

Figure S.2 ^{13}C-NMR Spectrum of 3,4-bis(((1R,2S)-2-(piperidin-1-yl)cyclohexyl) amino)cyclo but-3-ene-1,2-dione (**I**)

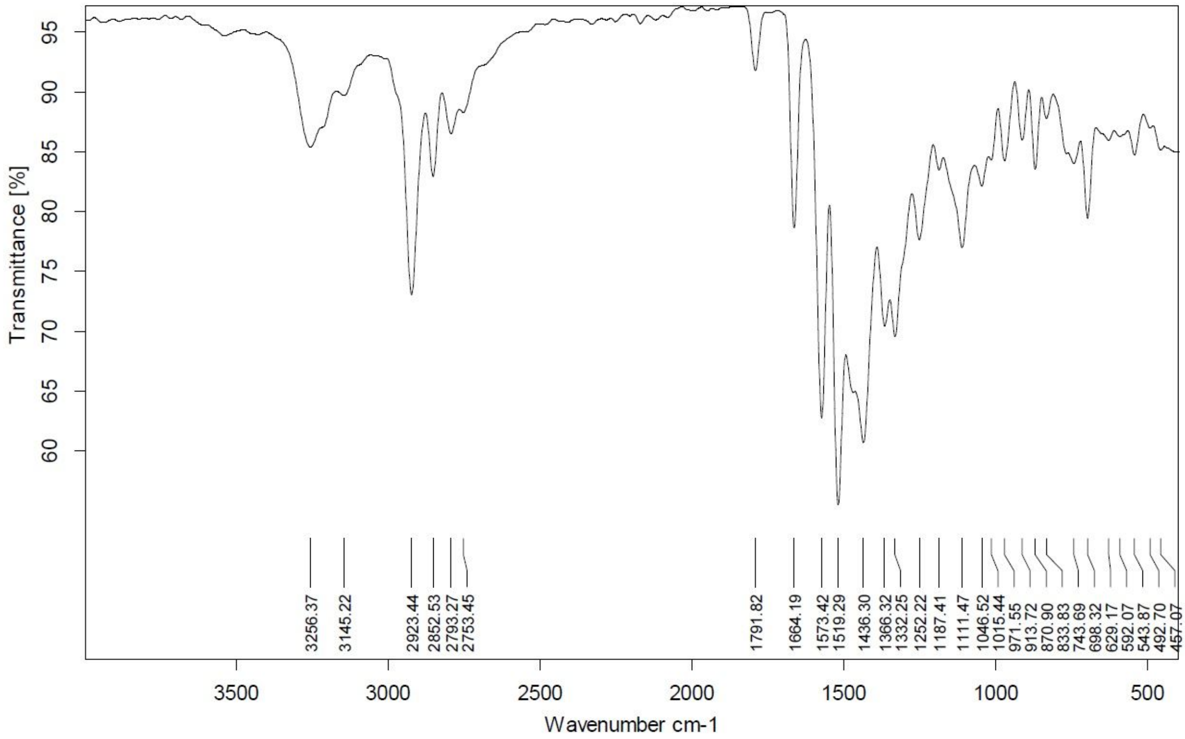

Figure S3. ATR-FTIR spectrum of 3-methoxy-4-((4-(trifluoromethyl)phenethyl)amino) cyclobut-3-ene-1,2-dione (**I**)

Qualitative Analysis Report

**Data Filename**	Sample25.d	**Sample Name**	Sample25
**Sample Type**	Sample	**Position**	P1-C7
**Instrument Name**	Instrument 1	**User Name**	Oguzhan DALKILIC
**Acq Method**	ESI pos.m	**Acquired Time**	12/14/2022 3:01:08 PM
**IRM Calibration Status**	Success	**DA Method**	Default.m
**Comment**			

**Sample Group**		**Info.**	
**Stream Name**	LC 1	**Acquisition SW Version**	6200 series TOF/6500 series Q-TOF B.08.00 (B8058.0)

User Chromatograms

Fragmentor Voltage 90 **Collision Energy** 0 **Ionization Mode** ESI

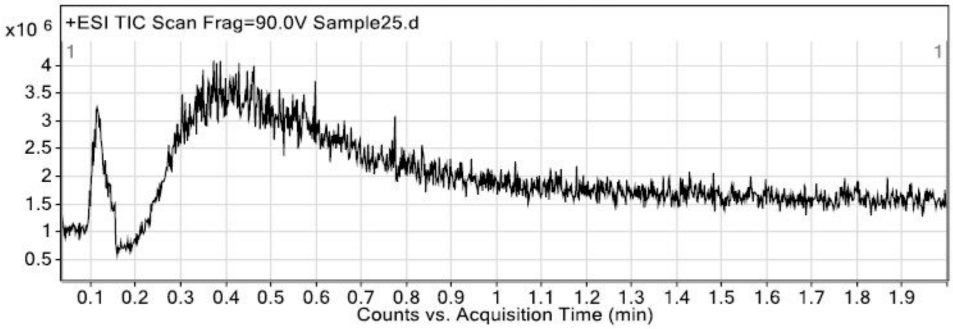

User Spectra

Fragmentor Voltage	Collision Energy	Ionization Mode
90	0	ESI

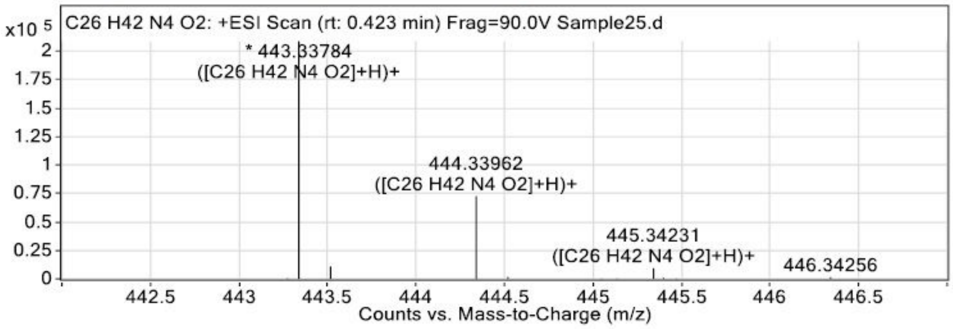

Peak List

m/z	z	Abund	Formula	Ion
443.33784	1	208657.42	C26 H42 N4 O2	(M+H)+
443.51638	1	10903.31		
444.33962	1	72442.04	C26 H42 N4 O2	(M+H)+
444.51909	1	1775.24		
445.34231	1	9396.26	C26 H42 N4 O2	(M+H)+

Formula Calculator Element Limits

Element	Min	Max
C	3	26
H	0	42
O	0	2
N	0	4

Formula Calculator Results

Formula	Best	Mass	Tgt Mass	Diff (ppm)	Ion Species	Score
C26 H42 N4 O2	DOĞRU	442,33019	442,33078	1,34	C26 H43 N4 O2	93,89

--- End Of Report ---

Figure S4. LC/MS-TOF Spectrum of 3,4-bis(((1R,2S)-2-(piperidin-1-yl)cyclohexyl)amino) cyclobut-3-ene-1,2-dione (**I**)

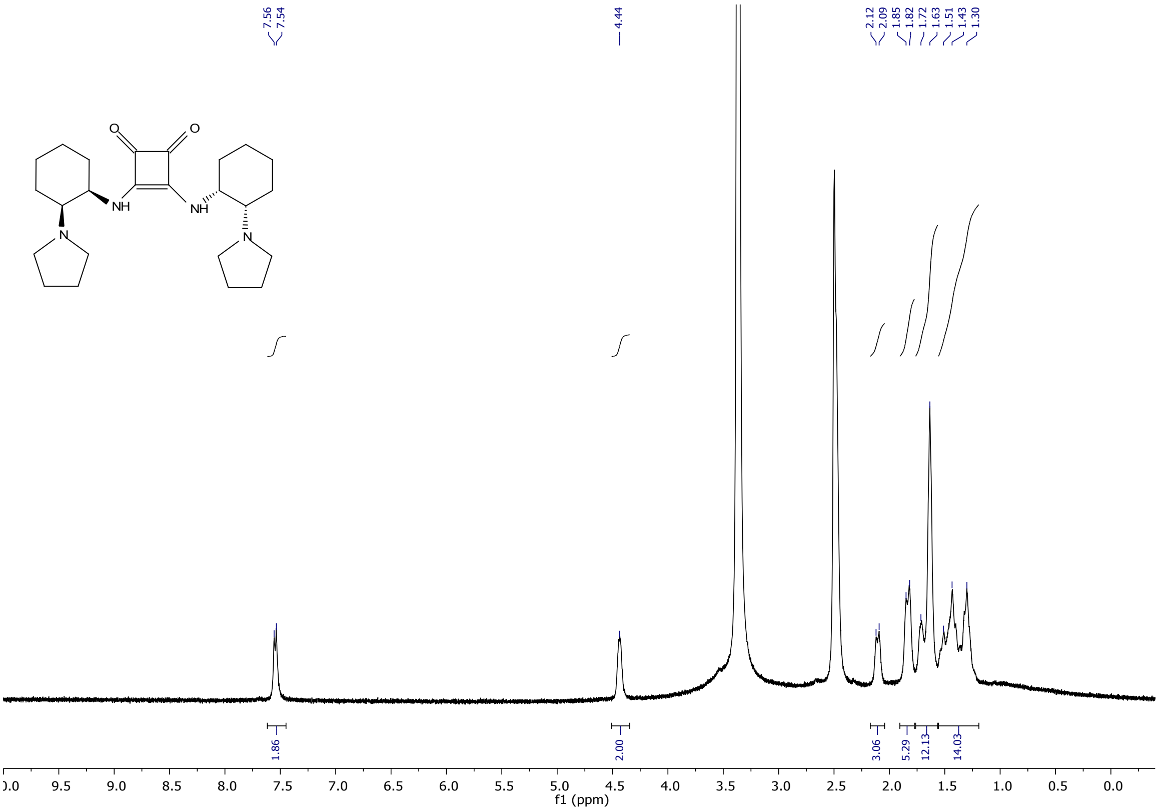

Figure S.5 ^{1}H-NMR Spectrum of 3,4-bis(((1R,2S)-2-(pyrrolidin-1-yl)cyclohexyl) amino)cyclo but-3-ene-1,2-dione (**II**)

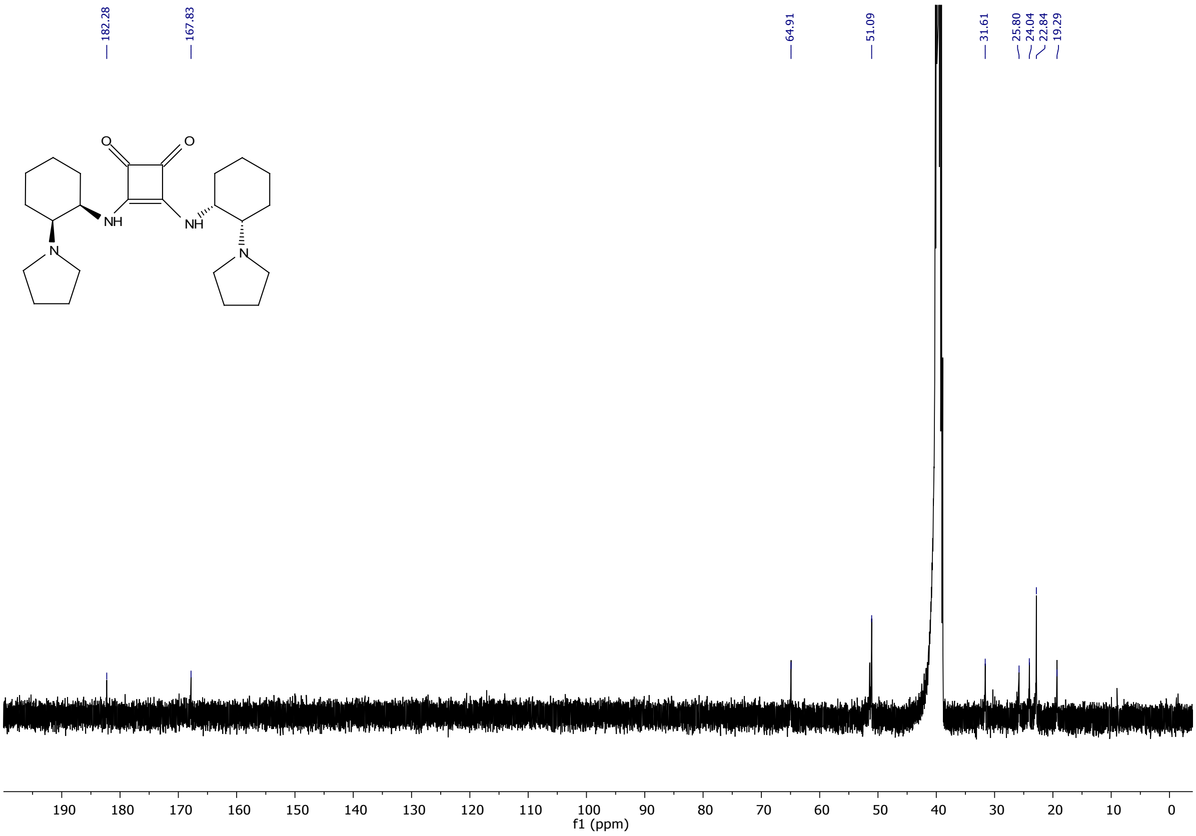

Figure S.6 ^{13}C-NMR Spectrum of 3,4-bis(((1R,2S)-2-(pyrrolidin-1-yl)cyclohexyl)amino) cyclo but-3-ene-1,2-dione (**II**)

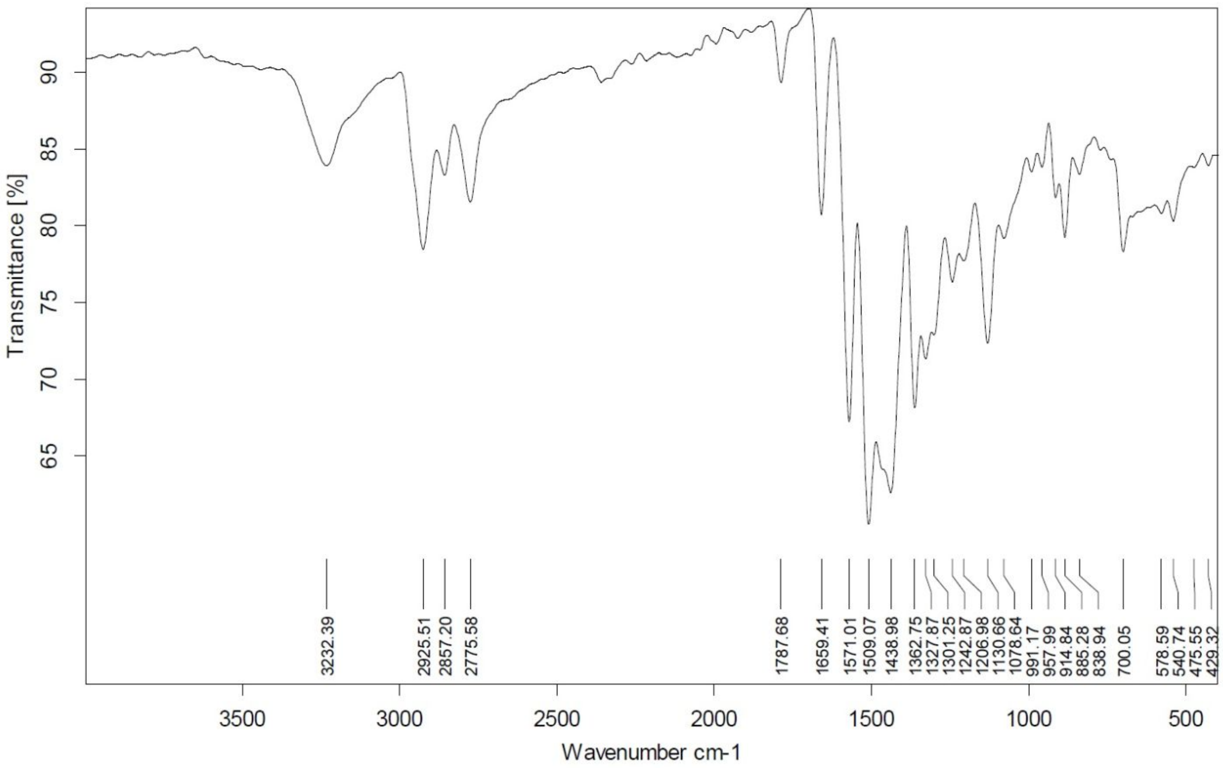

Figure S.7 ATR-FTIR Spectrum of 3,4-bis(((1R,2S)-2-(pyrrolidin-1-yl)cyclohexyl) amino) cyclo but-3-ene-1,2-dione (**II**)

Qualitative Analysis Report

**Data Filename**	Sample26.d	**Sample Name**	Sample26
**Sample Type**	Sample	**Position**	P1-C8
**Instrument Name**	Instrument 1	**User Name**	Oguzhan DALKILIC
**Acq Method**	ESI pos.m	**Acquired Time**	12/14/2022 3:03:51 PM
**IRM Calibration Status**	Success	**DA Method**	Default.m
**Comment**			

**Sample Group**		**Info.**	
**Stream Name**	LC 1	**Acquisition SW Version**	6200 series TOF/6500 series Q-TOF B.08.00 (B8058.0)

User Chromatograms

Fragmentor Voltage 90 **Collision Energy** 0 **Ionization Mode** ESI

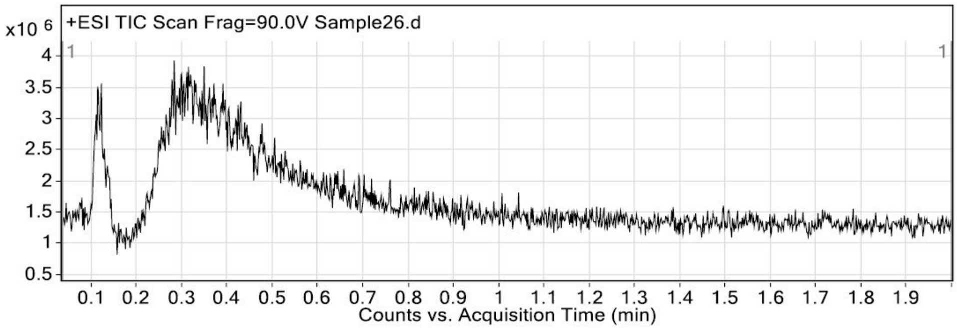

User Spectra

Fragmentor Voltage 90 **Collision Energy** 0 **Ionization Mode** ESI

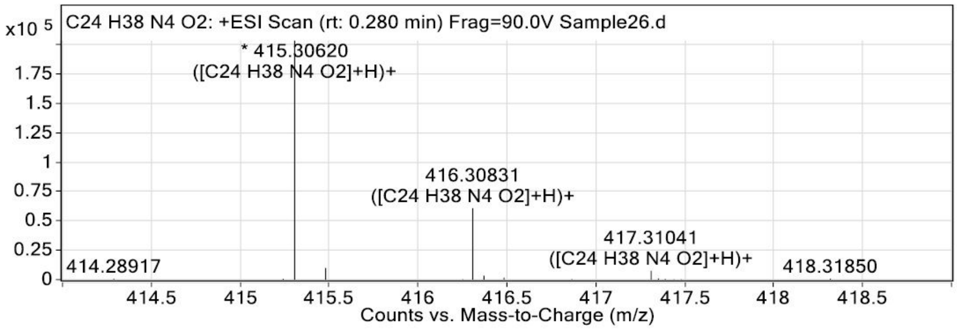

Peak List

m/z	z	Abund	Formula	Ion
415.3062	1	203171.59	C24 H38 N4 O2	(M+H)+
415.48018	1	9894.28		
416.30831	1	60697.04	C24 H38 N4 O2	(M+H)+
416.37109	2	3371.82		
416.48323	1	1624.92		
417.31041	1	7565.88	C24 H38 N4 O2	(M+H)+

Formula Calculator Element Limits

Element	Min	Max
C	3	24
H	0	38
O	0	2
N	0	4

Formula Calculator Results

Formula	Best	Mass	Tgt Mass	Diff (ppm)	Ion Species	Score
C24 H38 N4 O2	DOĞRU	414,29866	414,29948	1,96	C24 H39 N4 O2	96,29

--- End Of Report ---

Figure S8. LC/MS-TOF Spectrum of 3,4-bis(((1R,2S)-2-(pyrrolidin-1-yl)cyclohexyl)amino) cyclo but-3-ene-1,2-dione (**II**)

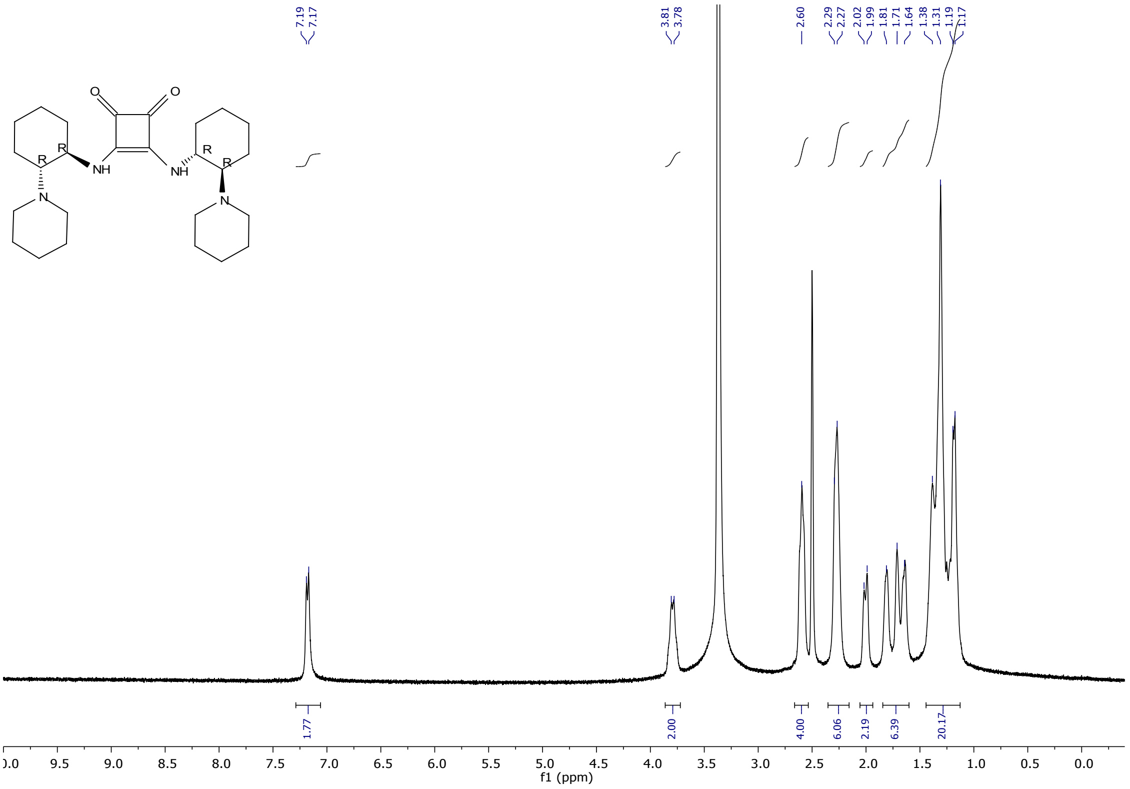

Figure S.9 ^{1}H-NMR Spectrum of 3,4-bis(((1R,2R)-2-(piperidin-1-yl)cyclohexyl)amino)cyclo but-3-ene-1,2-dione (**III**)

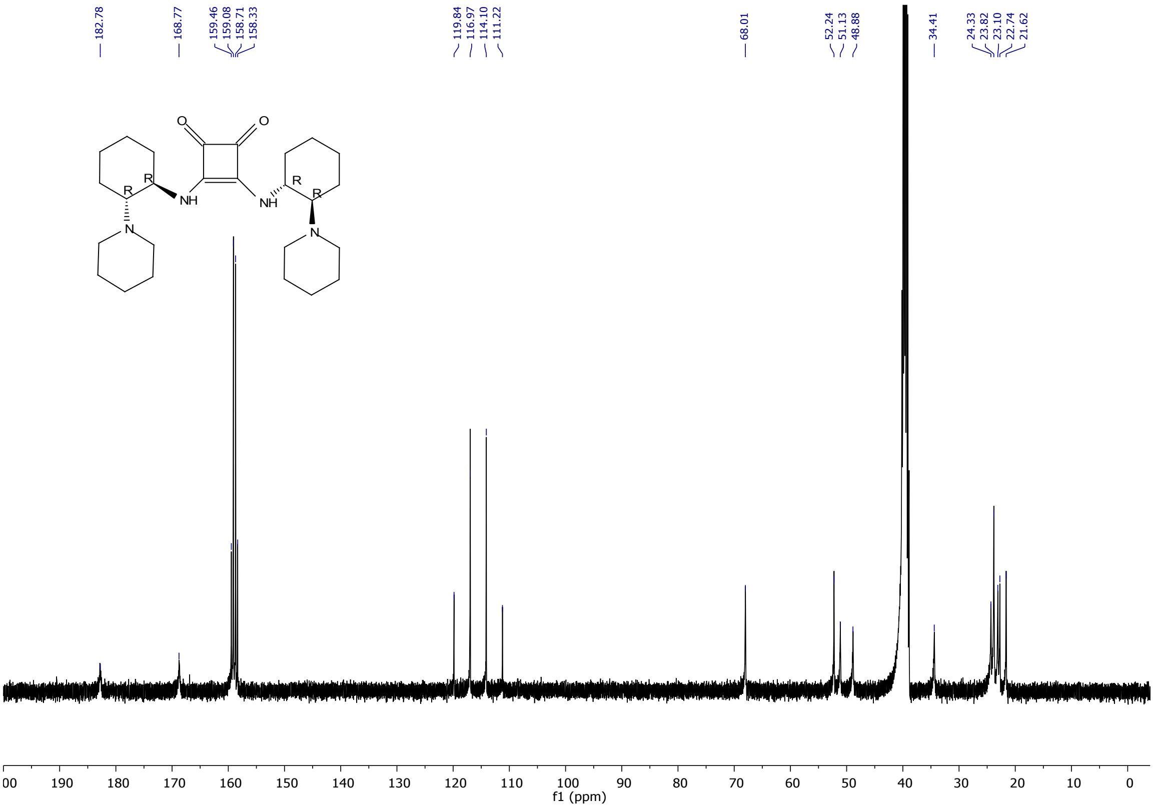

Figure S.10 ^{13}C-NMR Spectrum of 3,4-bis(((1R,2R)-2-(piperidin-1-yl)cyclohexyl)amino) cyclobut-3-ene-1,2-dione + **TFA**

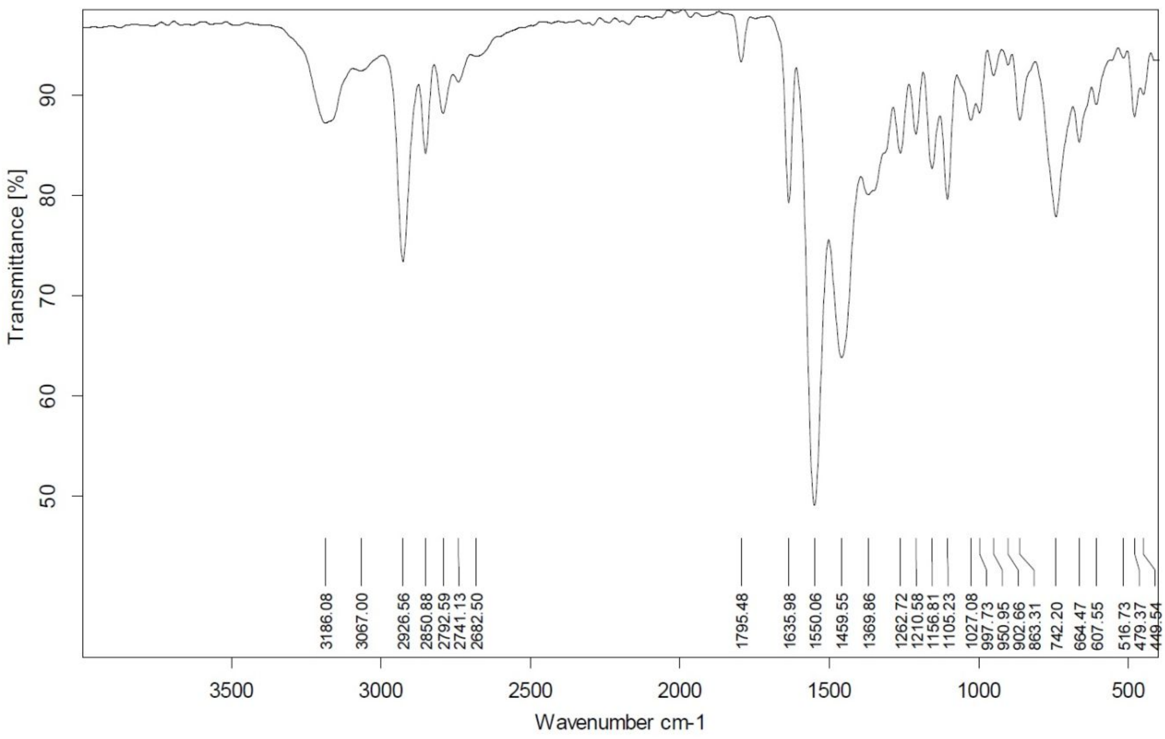

Figure S.11 ATR-FTIR Spectrum of 3,4-bis(((1R,2R)-2-(piperidin-1-yl)cyclohexyl) amino) cyclobut-3-ene-1,2-dione (**III**)

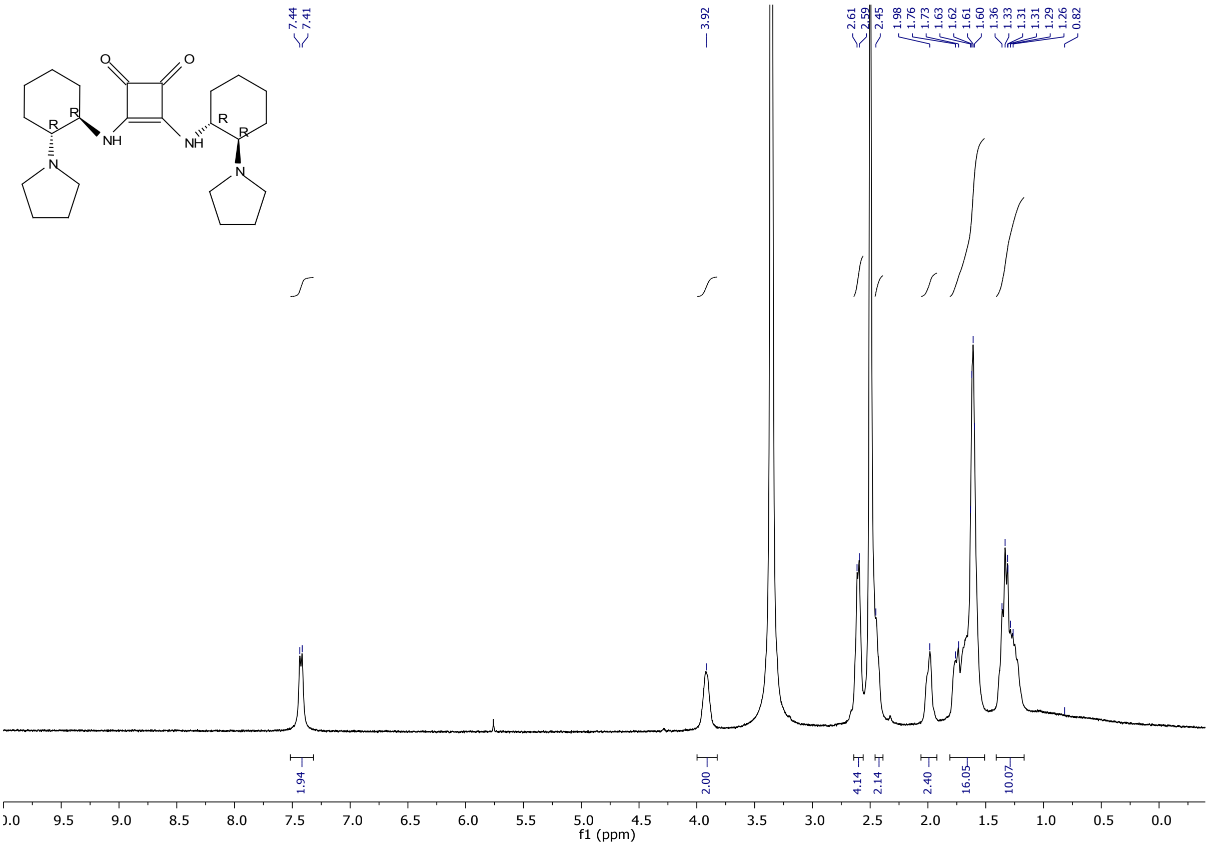

Figure S.12 ^{1}H-NMR Spectrum of 3,4-bis(((1R,2R)-2-(piperidin-1-yl)cyclohexyl)amino) cyclo but-3-ene-1,2-dione (**IV**)

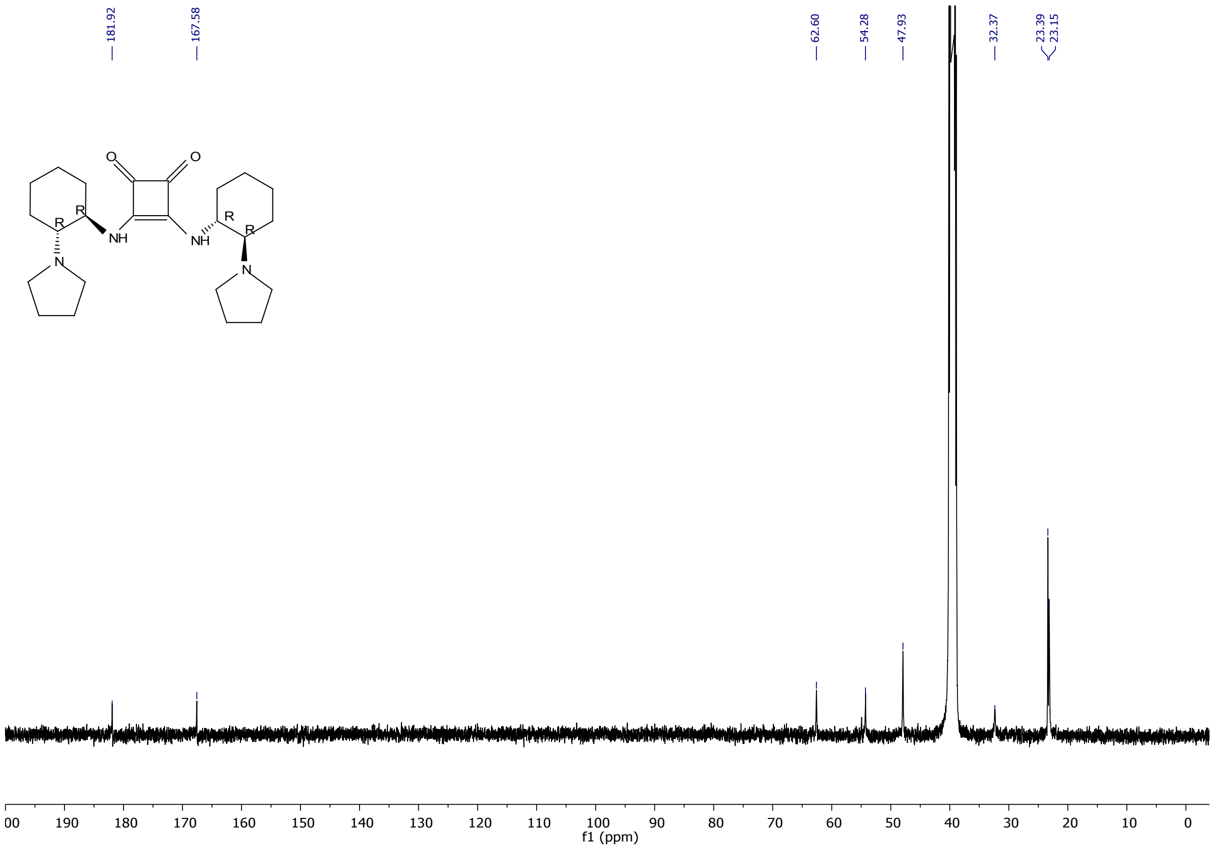

Figure S.13 ^{13}C-NMR Spectrum of 3,4-bis(((1R,2R)-2-(piperidin-1-yl)cyclohexyl) amino) cyclo but-3-ene-1,2-dione (**IV**)

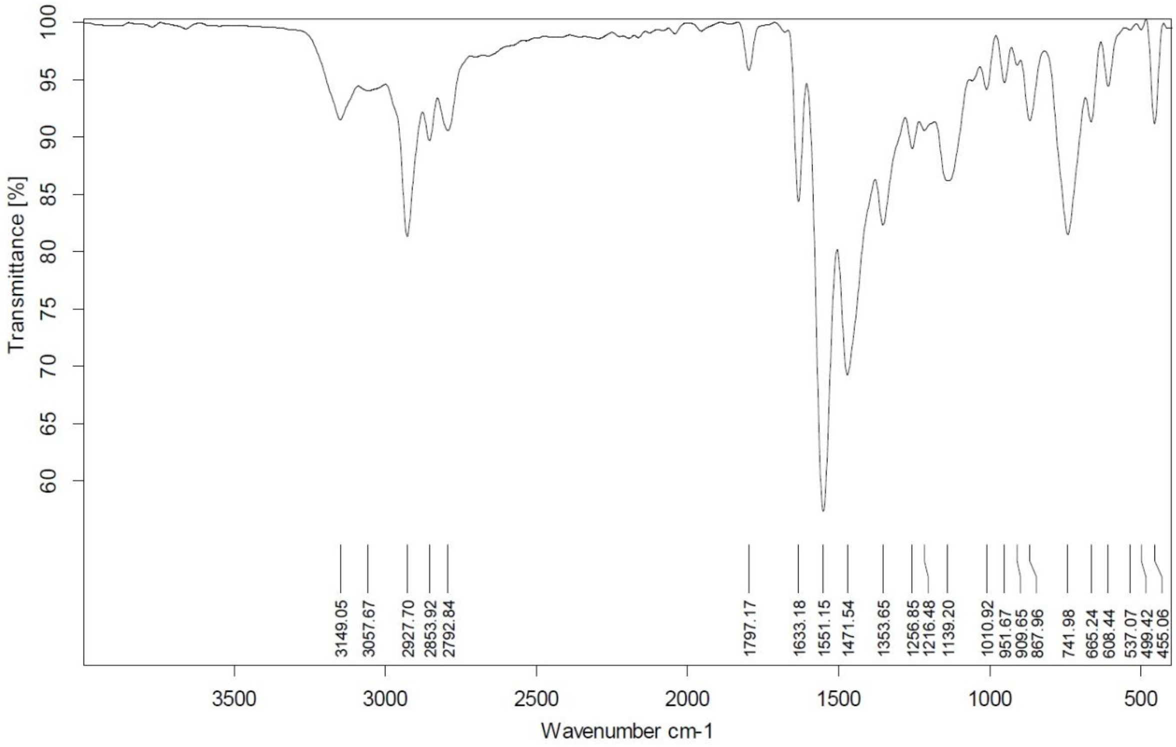

Figure S.14 ATR-FTIR Spectrum of 3,4-bis(((1R,2R)-2-(piperidin-1-yl)cyclohexyl)amino) cyclo but-3-ene-1,2-dione (**IV**)

Qualitative Analysis Report

**Data Filename**	Sample24.d	**Sample Name**	Sample24
**Sample Type**	Sample	**Position**	P1-C6
**Instrument Name**	Instrument 1	**User Name**	Oguzhan DALKILIC
**Acq Method**	ESI pos.m	**Acquired Time**	12/14/2022 2:58:25 PM
**IRM Calibration Status**	Success	**DA Method**	Default.m
**Comment**			

**Sample Group**		**Info.**	
**Stream Name**	LC 1	**Acquisition SW Version**	6200 series TOF/6500 series Q-TOF B.08.00 (B8058.0)

User Chromatograms

Fragmentor Voltage 90 **Collision Energy** 0 **Ionization Mode** ESI

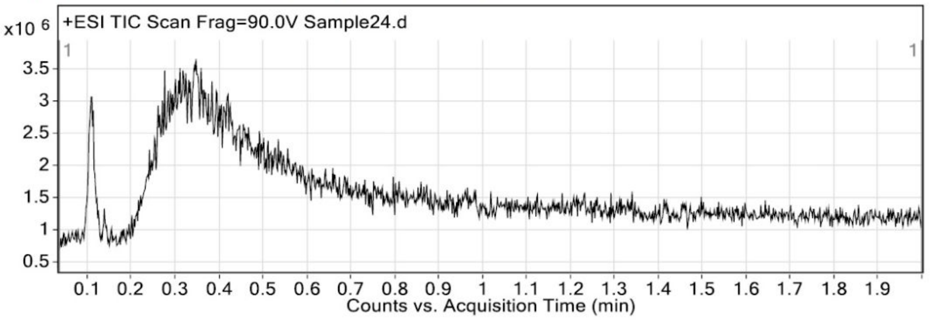

User Spectra

Fragmentor Voltage 90 **Collision Energy** 0 **Ionization Mode** ESI

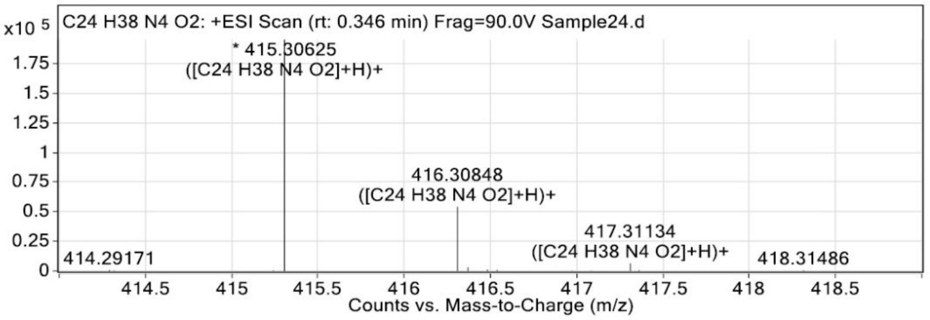

Peak List

m/z	z	Abund	Formula	Ion
415.30625	1	195122.58	C24 H38 N4 O2	(M+H)+
416.30848	1	54138.96	C24 H38 N4 O2	(M+H)+
416.36965	1	3043.63		
416.48285	2	1261.86		
416.53862	2	951.36		
417.31134	1	6410.17	C24 H38 N4 O2	(M+H)+

Formula Calculator Element Limit

Element	Min	Max
C	3	24
H	0	38
O	0	2
N	0	4

Formula Calculator Results

Formula	Best	Mass	Tgt Mass	Diff (ppm)	Ion Species	Score
C24 H38 N4 O2	DOĞRU	414,29877	414,29948	1,7	C24 H39 N4 O2	97,75

--- End Of Report ---

Figure S15. LC/MS-TOF Spectrum of 3,4-bis(((1R,2R)-2-(piperidin-1-yl)cyclohexyl) amino) cyclo but-3-ene-1,2-dione (**IV**)

II. Stack plots of ^{1}H NMR spectra of addition of TBA-Br to SQs II-IV

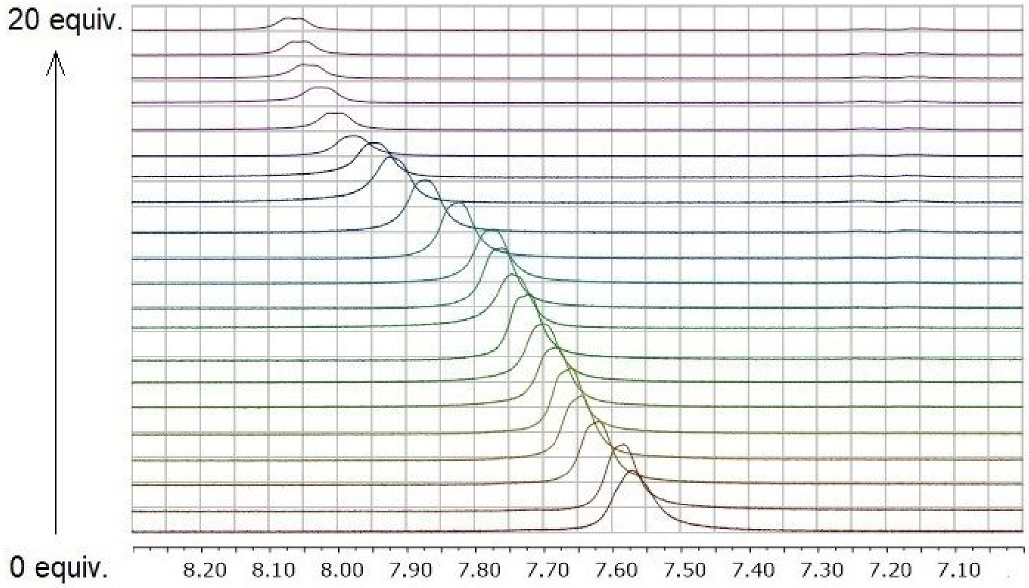

Figure S16. Stack plots of ^{1}H NMR spectra of addition of TBA-Br (0–20 equiv) in <0.02% H_2O in [d_6] DMSO at 25 ^{o}C to squaramide **II**

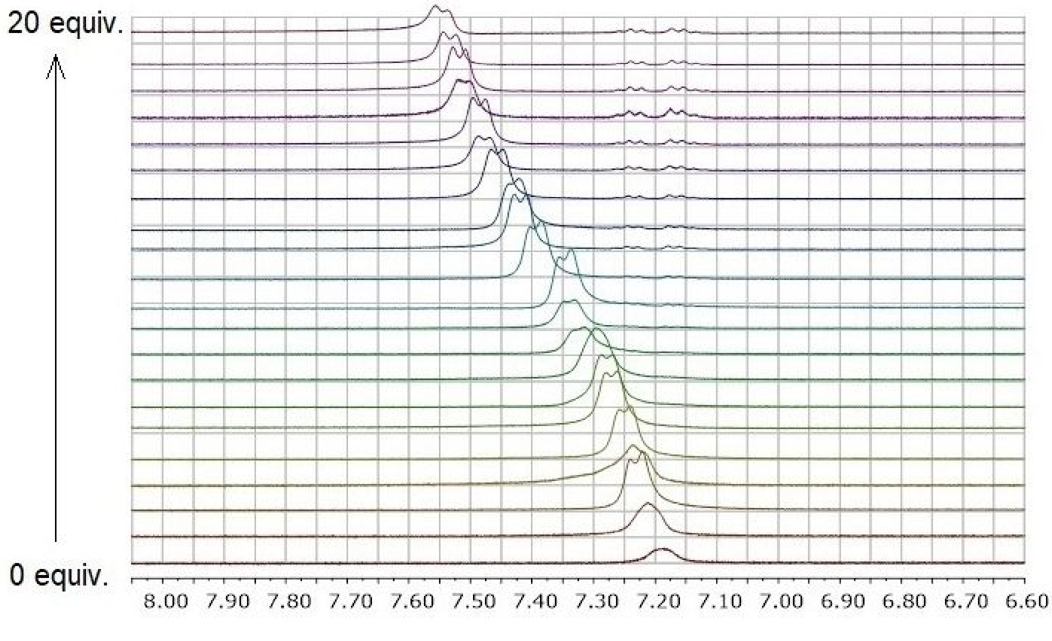

Figure S17. Stack plots of ^{1}H NMR spectra of addition of TBA-Br (0–20 equiv) in <0.02% H_2O in [d_6] DMSO at 25 ^{o}C to squaramide **III**

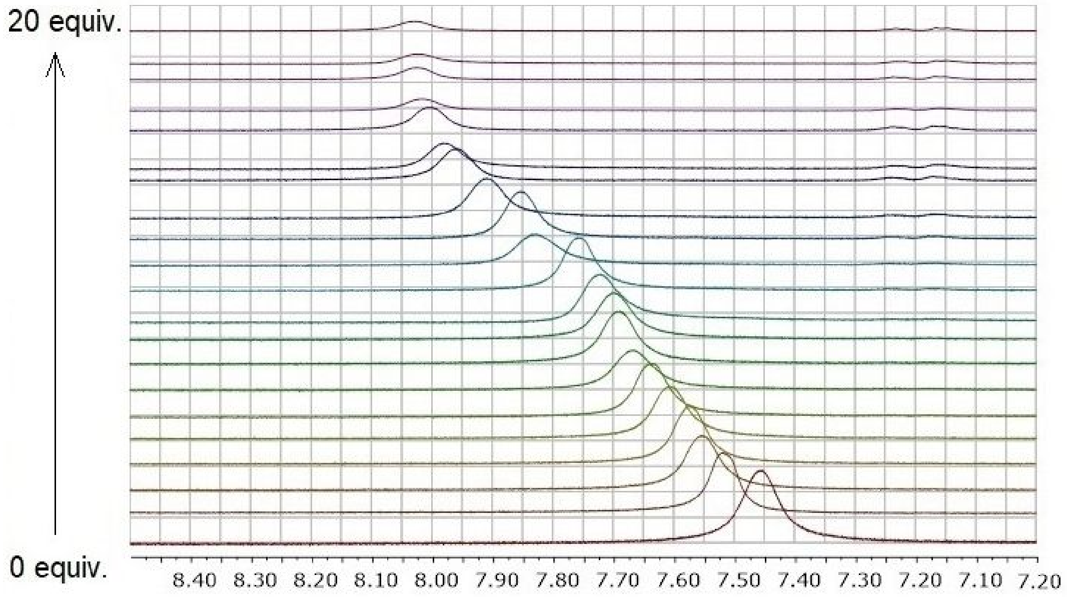

Figure S18. Stack plots of ^{1}H NMR spectra of addition of TBA-Br (0–20 equiv) in <0.02% H_2O in [d$_6$] DMSO at 25 ^{o}C to squaramide **IV**

III. Example 1:1 Binding Model DYNAFIT Script[1]

```
[task]
   data = equilibria
   task = fit

[mechanism]
   H + G <==> H.G      :       Ka    assoc

[constants]
   Ka =  ??

[responses]
   intensive ; ... NMR chemical shifts

[data]
   variable G, H
   plot titration ; ... [H] and [G] are varied simultaneously
   graph 1H ; proton chemical shifts
   set aH | response H = 7.47 ?, H.G = 8.94 ?

[output]
   directory ./examples/pt03/NMR/output/aa
[settings]
{Output
   XAxisLabel = [Guest], M
   YAxisLabel = chemical shift, ppm
[end]
```

IV. Graphs of DynaFit 1:1 Analysis[1]

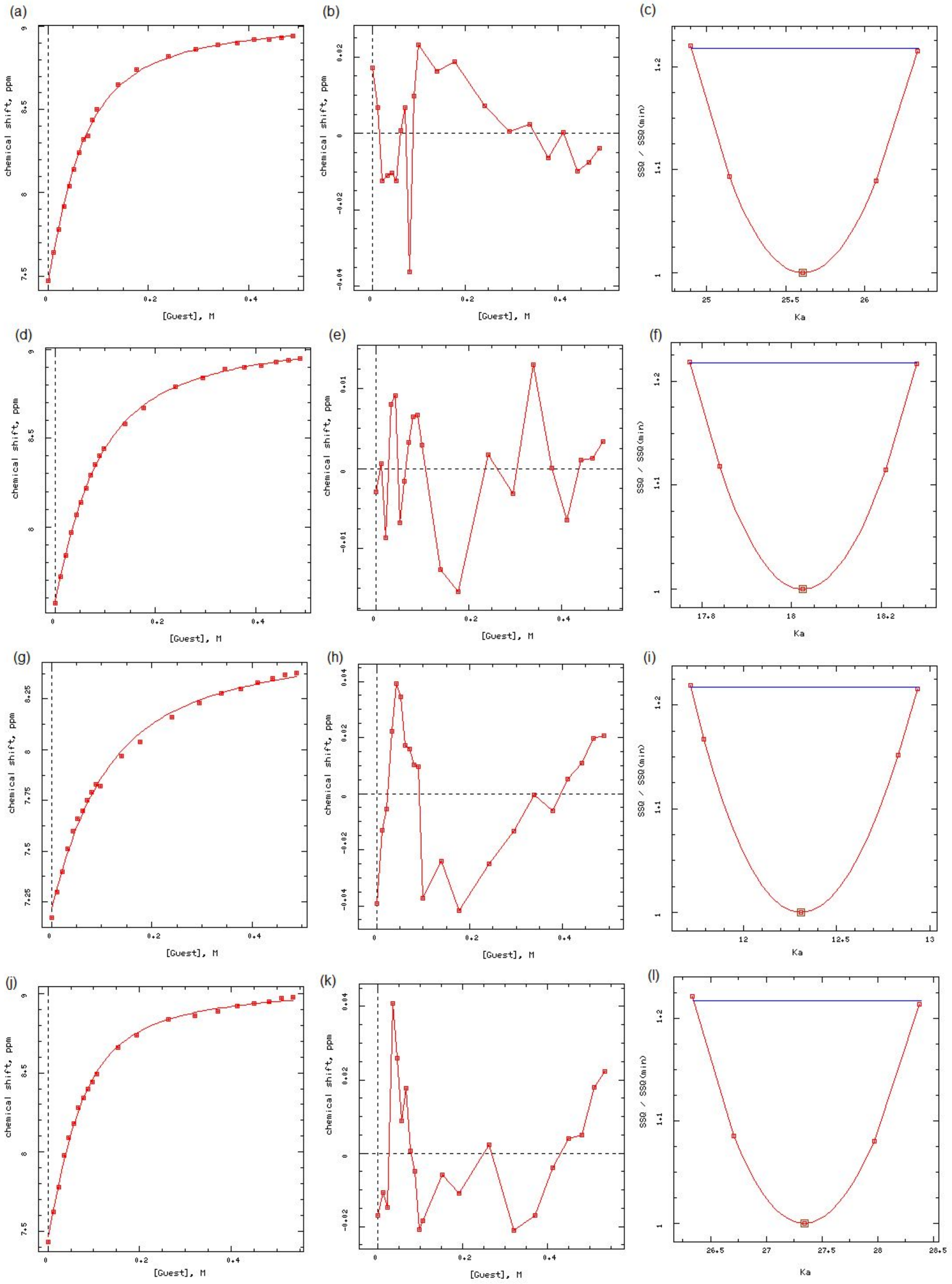

Figure S19. Plot of downfield shifts of NH protons versus TBA-Cl concentration according to DynaFit 1:1 Analysis during ^{1}H NMR titration in d_6-DMSO at (**a**) δ = 7.47 ppm in Sq. **I**, **(d)** δ = 7.57 ppm in Sq. **II**, **(g)** δ = 7.17 ppm in Sq. **III**, **(j)** δ = 7.43 ppm in Sq. **IV**; Residual **(b)** δ = 7.47 ppm in Sq. **I** **(e)** δ = 7.57 ppm in Sq. **II** **(h)** δ = 7.17 ppm in Sq. **III** **(k)** δ = 7.43 ppm in Sq. **IV**; Relative sum of squares (SSQ/SSQ min.) **(c)** K_a =

25.6087 M^{-1} (NH-proton at δ = 7.47 ppm), **(f)** K_a = 18.0244 M^{-1} (NH-proton at δ = 7.57 ppm), **(i)** K_a = 12.3113 M^{-1} (NH-proton at δ = 7.17 ppm), **(l)** K_a = 27.3393 M^{-1} (NH-proton at δ = 7.43 ppm).

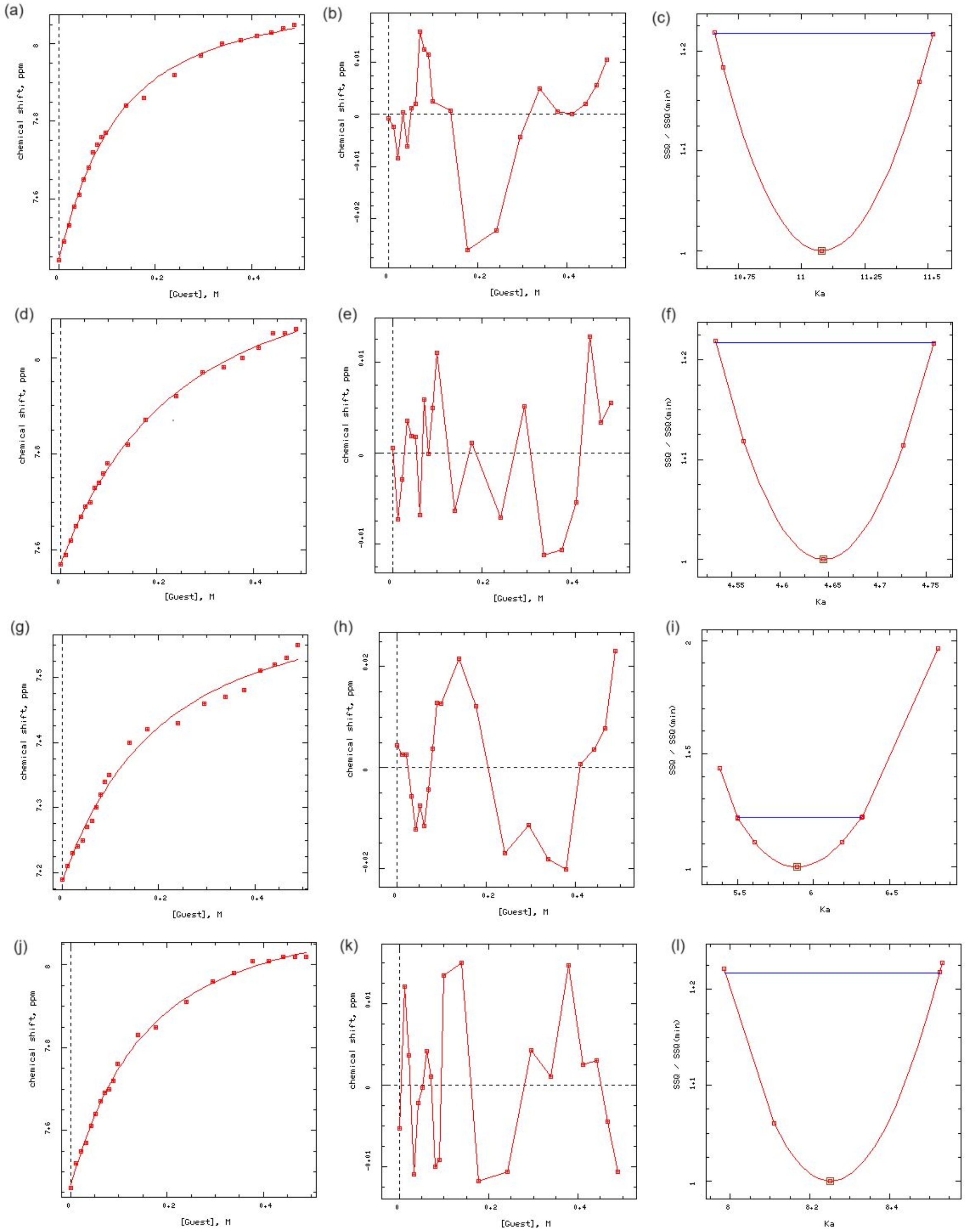

Figure S20. Plot of downfield shifts of NH protons versus TBA-Br concentration according to DynaFit 1:1 Analysis[1] during ^{1}H NMR titration in d_6-DMSO at **(a)** δ = 7.47 ppm in Sq. **I**, **(d)** δ = 7.57 ppm in Sq. **II**, **(g)** δ = 7.17 ppm in Sq. **III**, **(j)** δ = 7.43 ppm in Sq. **IV**; Residual **(b)** δ = 7.47 ppm in Sq. **I** **(e)** δ = 7.57 ppm in Sq. **II** **(h)** δ = 7.17 ppm in Sq. **III** **(k)** δ = 7.43 ppm in Sq. **IV**; Relative sum of squares (SSQ/SSQ min.) **(c)** K_a =

11.0800 M^{-1} (NH-proton at δ = 7.47 ppm), **(f)** K_a = 4.6447 M^{-1} (NH-proton at δ = 7.57 ppm), **(i)** K_a = 5.8912 M^{-1} (NH-proton at δ = 7.17 ppm), **(l)** K_a = 8.2511 M^{-1} (NH-proton at δ = 7.43 ppm).

V. Graphs of BindFit 1:1 analysis[2, 3]

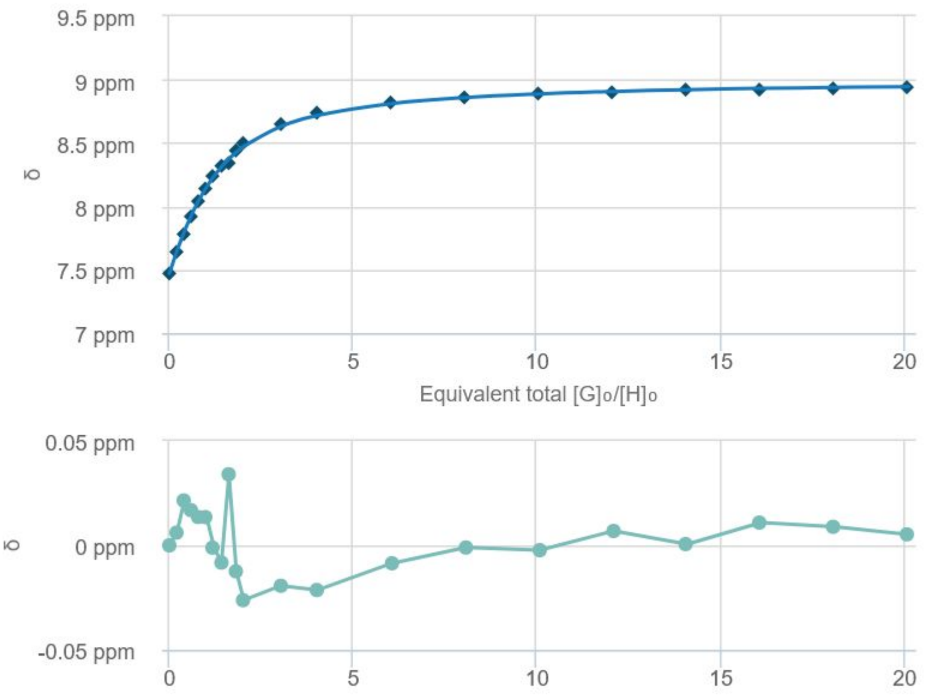

Figure S21. Fitplot for NH proton at δ = 7.47 ppm. K_a = 24.63 M^{-1} and Residual according to BindFit 1:1 analysis of ^{1}H NMR titration between Sq. **I** and TBA-Cl

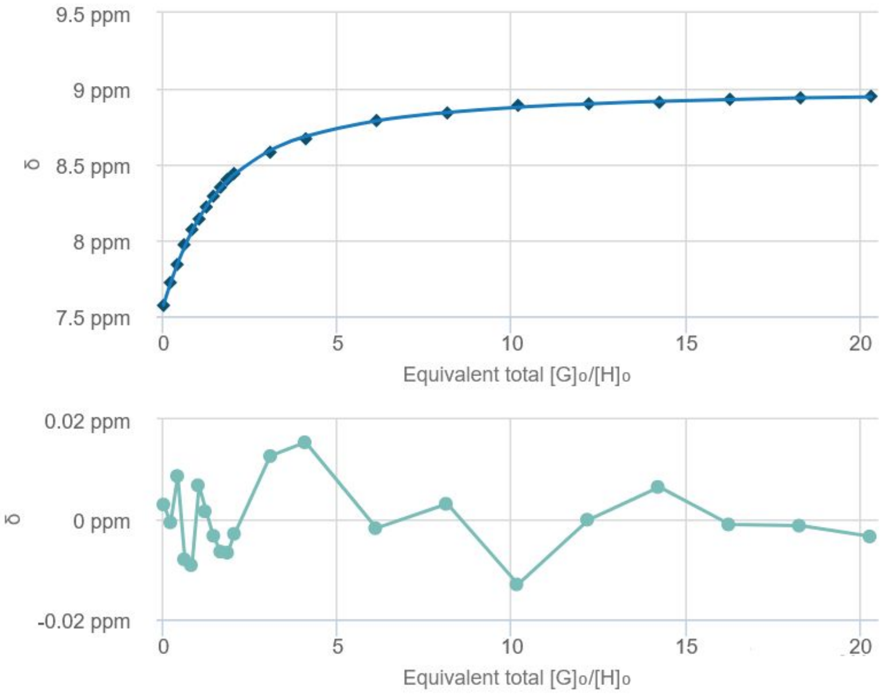

Figure S22. Fitplot for NH proton at δ = 7.57 ppm. K_a = 18.02 M^{-1} and Residual according to BindFit 1:1 analysis of ^{1}H NMR titration between Sq. **II** and TBA-Cl

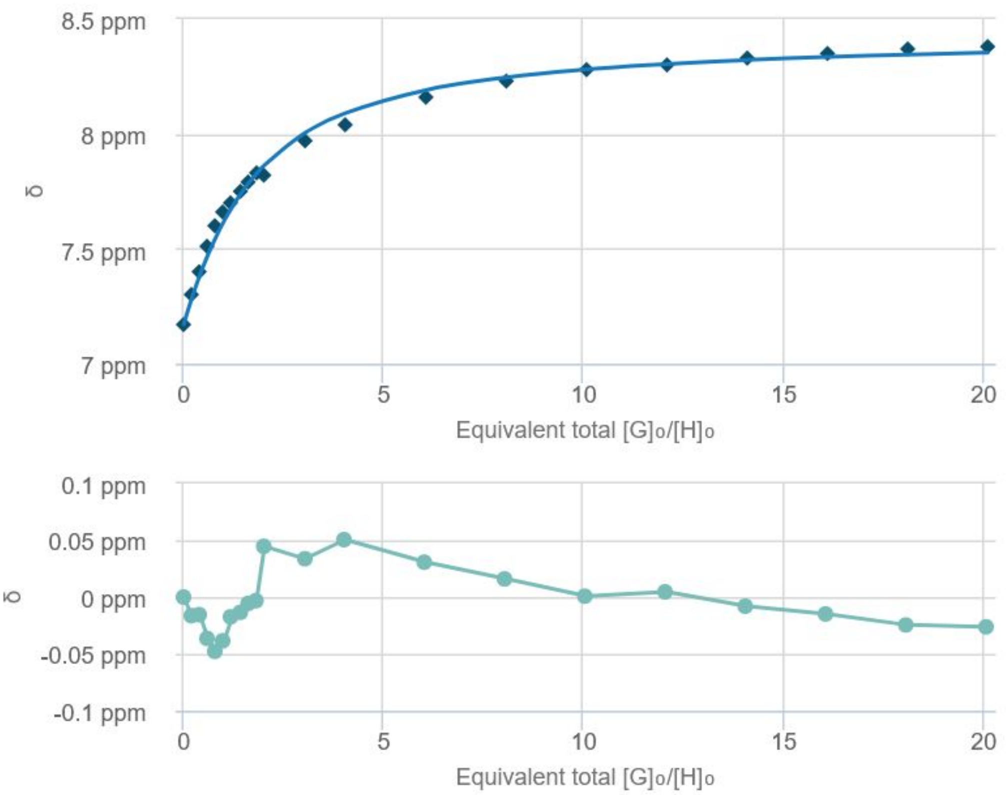

Figure S23. Fitplot for NH proton at δ = 7.17 ppm. K_a = 14.13 M^{-1} and Residual according to BindFit 1:1 analysis of ^{1}H NMR titration between Sq. **III** and TBA-Cl

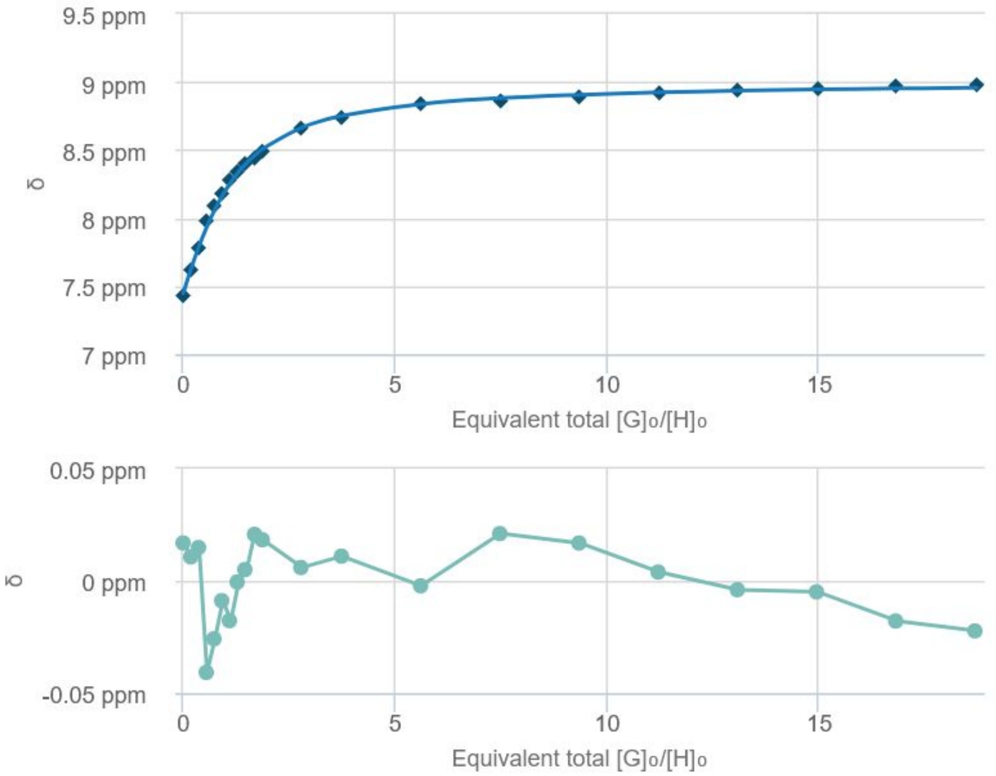

Figure S24. Fitplot for NH proton at δ = 7.43 ppm. K_a = 27.34 M^{-1} and Residual according to BindFit 1:1 analysis of ^{1}H NMR titration between Sq. **IV** and TBA-Cl

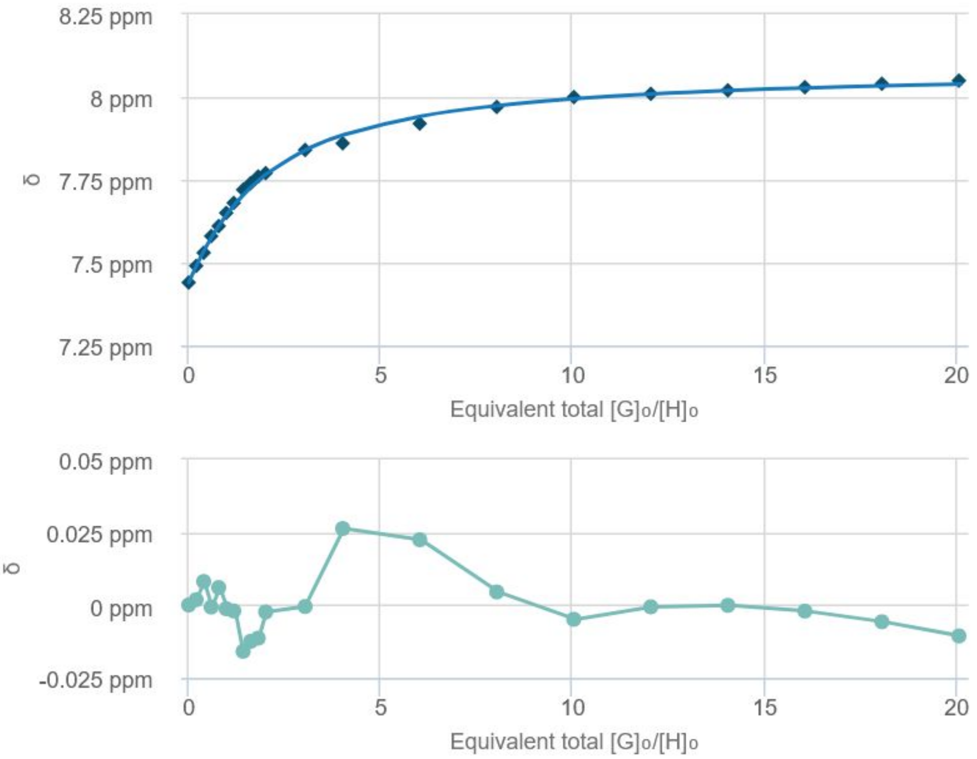

Figure S25. Fitplot for NH proton at δ = 7.47 ppm. K_a = 11.14 M^{-1} and Residual according to BindFit 1:1 analysis of ^{1}H NMR titration between Sq. **I** and TBA-Br

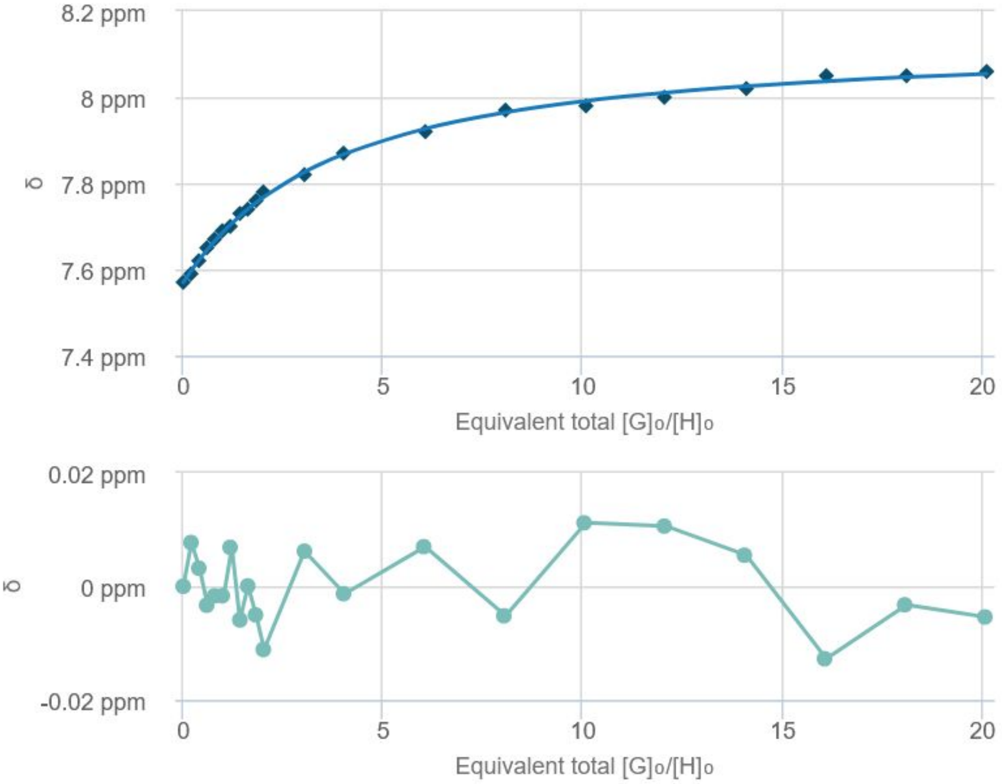

Figure S26. Fitplot for NH proton at δ = 7.57 ppm. K_a = 4.63 M^{-1} and Residual according to BindFit 1:1 analysis of ^{1}H NMR titration between Sq. **II** and TBA-Br

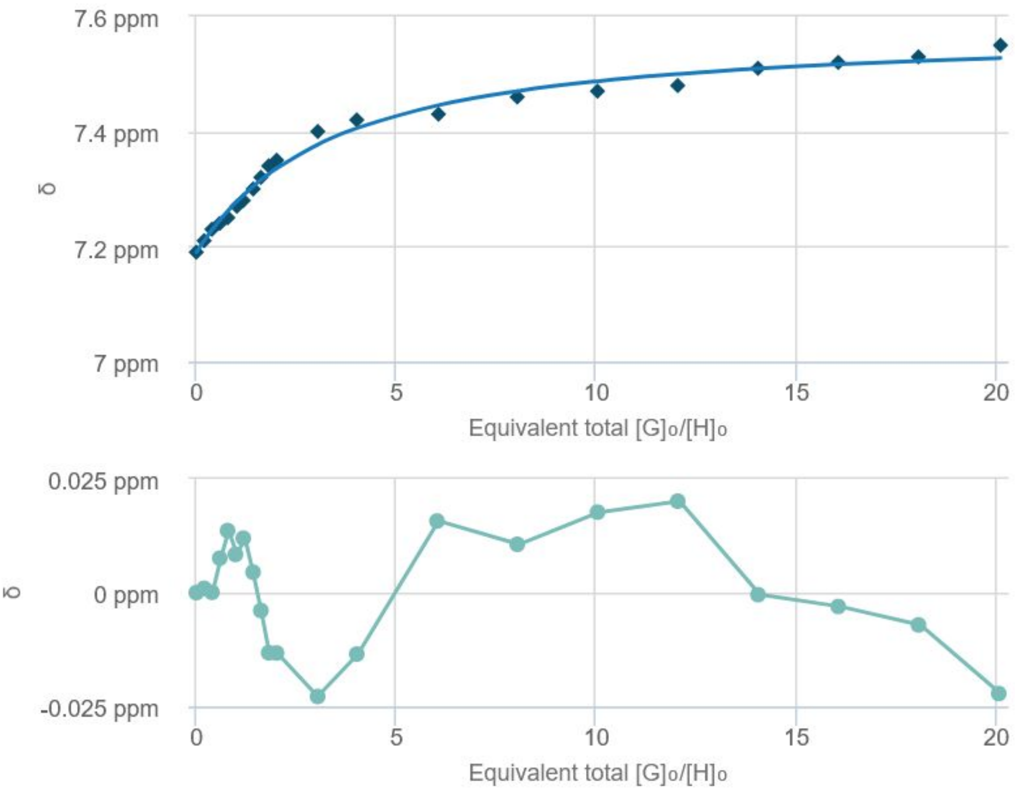

Figure S27. Fitplot for NH proton at δ = 7.17 ppm. K_a = 5.49 M^{-1} and Residual according to BindFit 1:1 analysis of ^1H NMR titration between Sq. **III** and TBA-Br

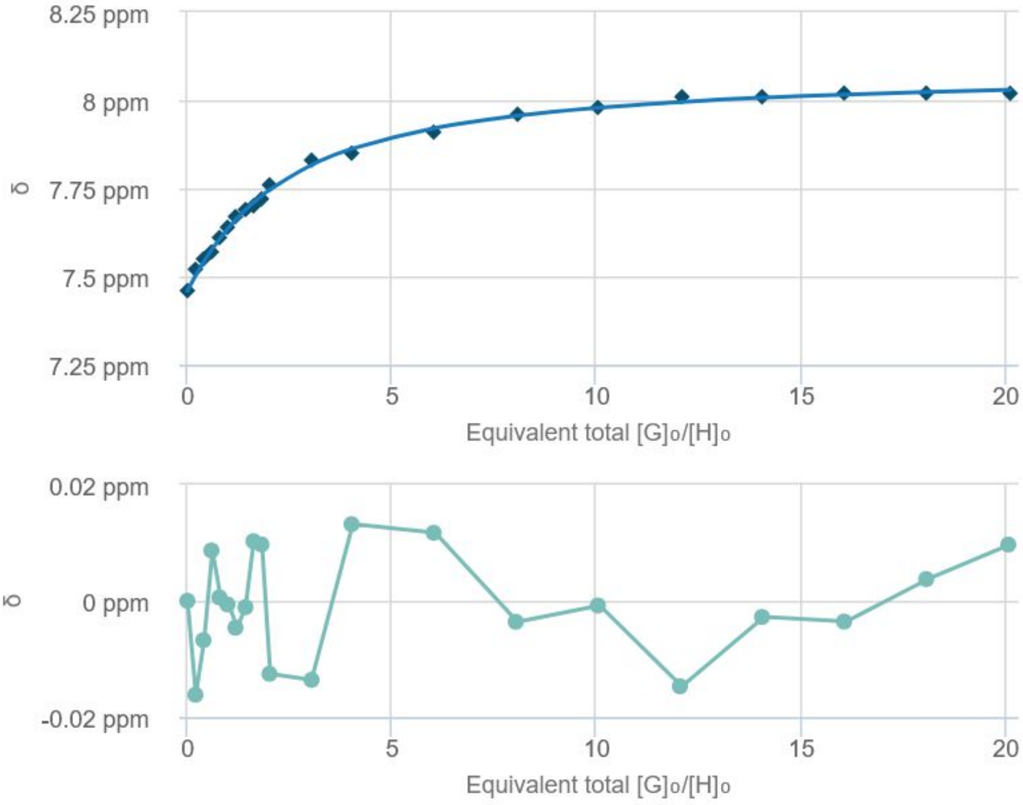

Figure S28. Fitplot for NH proton at δ = 7.43 ppm. K_a = 8.64 M^{-1} and Residual according to BindFit 1:1 analysis of ^1H NMR titration between Sq. **IV** and TBA-Br

VI. Job plots[4]

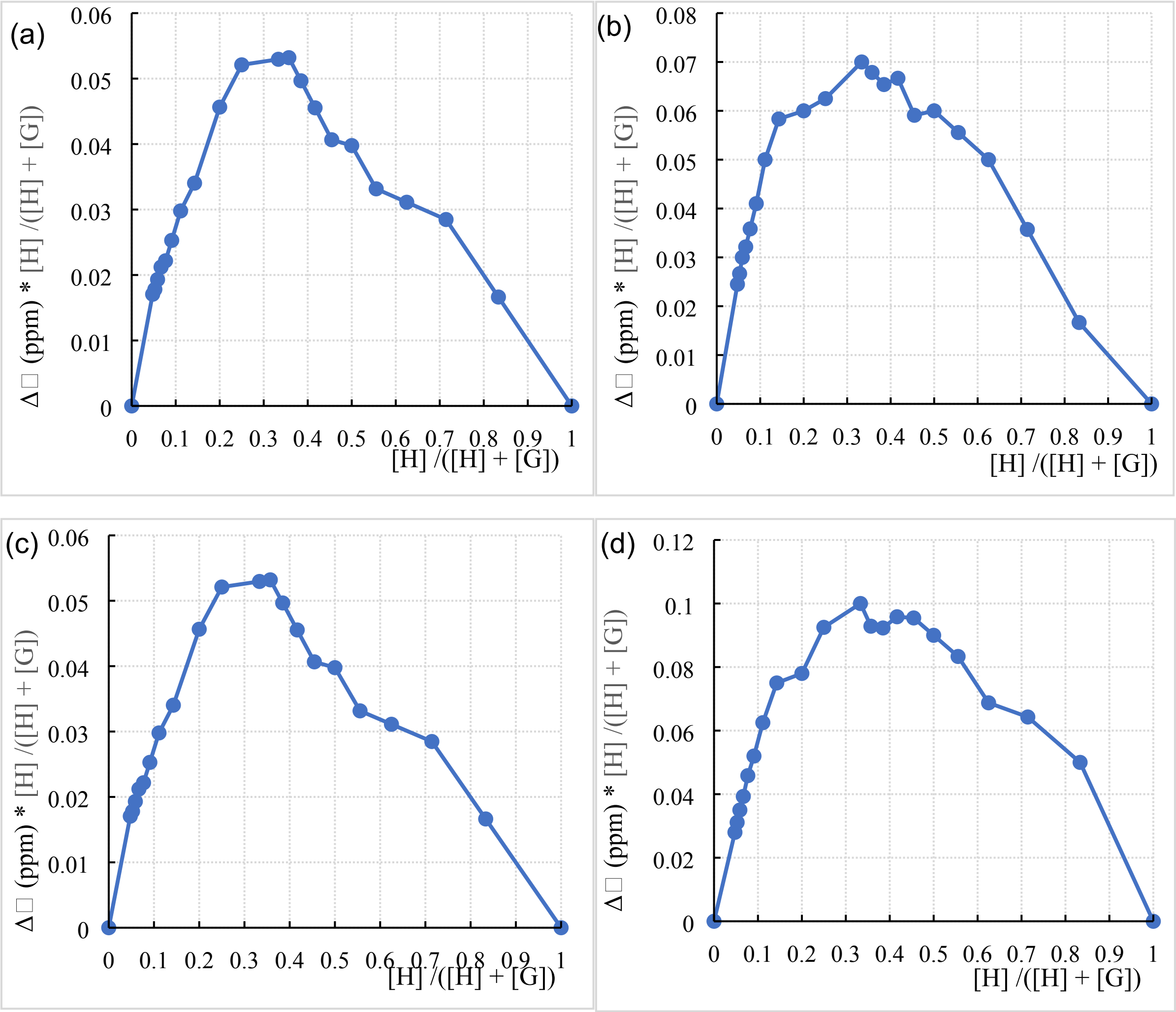

Figure S29. Job plots of ^{1}H-NMR spectroscopic TBA-Br titration with SQ **I** (X_{max}: 0.42) (**a**), SQ **II** (X_{max}: 0.33) (**b**), SQ **III** (X_{max}: 0.38) (**c**), SQ **IV** (X_{max}: 0.33) (**d**)

In the DynaFit script, both the host and guest concentration in the titration and all of chemical shift values in ppm are entered as table and a set of chemical equations as text[1, 5] in script as shown in detail in Supporting Information page S14. The DynaFit package automatically calculates each of the underlying mathematical information, taking binding and gradual dissociation into account.

In BindFit,[3] only the host and guest concentration in the titration and all chemical shift values in ppm are entered as excel data. After selecting the stoichiometric ratio, the program performs a non-linear regression on this data using the full binding equation implemented through a python program on BindFit.

REFERENCES

1. Kuzmic, P., DynaFit-a software package for enzymology. *Methods Enzymol.* **2009,** *467*, 247-280.
2. Busschaert, N.; Kirby, I. L.; Young, S.; Coles, S. J.; Horton, P. N.; Light, M. E.; Gale, P. A., Squaramides as Potent Transmembrane Anion Transporters. *Angew. Chem. Int. Ed.* **2012,** *51*, 4426-4430.
3. Brynn Hibbert, D.; Thordarson, P., The death of the Job plot, transparency, open science and online tools, uncertainty estimation methods and other developments in supramolecular chemistry data analysis. *Chem. Commun.* **2016,** *52* (87), 12792-12805.
4. Usacheva, T. R.; Volynkin, V. A.; Panyushkin, V. T.; Lindt, D. A.; Pham, T. L.; Nguyen, T. T. H.; Le, T. M. H.; Alister, D. A.; Kabirov, D. N.; Kuranova, N. N.; Gamov, G. A.; Kushnir, R. A.; Biondi, M.; Giancola, C.; Sharnin, V. A., Complexation of Cyclodextrins with Benzoic Acid in Water-Organic Solvents: A Solvation-Thermodynamic Approach. *Molecules* **2021,** *26* (15), 4408-4433.
5. Gasa, T. B.; Spruell, J. M.; Dichtel, W. R.; Sorensen, T. J.; Philp, D.; Stoddart, J. F.; Kuzmic, P., Complexation between methyl viologen (paraquat) bis(hexafluorophosphate) and dibenzo[24]crown-8 revisited. *Chemistry* **2009,** *15* (1), 106-116.
